# Body Size Decline in an Endangered Bat Is Associated With Climate Change at a Continental Scale but Varies by Phenophase and Region

**DOI:** 10.1111/gcb.70983

**Published:** 2026-07-14

**Authors:** Valerie von Zuben, Hugh G. Broders, Thomas S. Jung, Cori L. Lausen, Kaleigh J. O. Norquay, Craig K. R. Willis, Christina M. Davy

**Affiliations:** ^1^ Wildlife Research and Monitoring Section Ontario Ministry of Natural Resources Peterborough Canada; ^2^ Department of Biology University of Waterloo Waterloo Canada; ^3^ Department of Environment Government of Yukon Whitehorse Canada; ^4^ Department of Renewable Resources University of Alberta Edmonton Canada; ^5^ Western Canada Bat Program Wildlife Conservation Society Canada Kaslo Canada; ^6^ Department of Biology University of Winnipeg Winnipeg Canada; ^7^ Department of Biology Carleton University Ottawa Canada

**Keywords:** carry‐over effect, climate change, growth, little brown bat, *Myotis lucifugus*, nutritional stress

## Abstract

At high latitudes, hibernating species must replenish their energy reserves within a 4–6 month active season. During this window, insectivorous bats at higher latitudes must consume sufficient arthropod prey to support reproduction and migration, despite suboptimal weather and prey abundance that varies across time and space. Local declines in body size of endangered little brown bats ( *Myotis lucifugus* ) suggest ongoing nutritional stress associated with limited prey availability and inclement weather. We tested the hypothesis that climate change has caused nutritional stress in bats at a continental scale. We analyzed morphometric data from > 21,000 bats from five regions across Canada and northern Montana over 8–16 years. We used forearm length as a proxy for access to nutrition during development, and body mass as a proxy for recent energy reserves. Average adult and juvenile forearm length declined over time in most study regions, although the magnitude of the estimated declines varied geographically. We found that the interactive effects of rainfall and temperature on reproductive females exerted a carry‐over effect on juvenile body size, as juvenile forearm length was shorter, on average, in years when mothers experienced poor weather before and after hibernation. Although changes in adult body mass were geographically widespread, the drivers, direction, and magnitude of these trends varied among phenophases, demographic groups, and study regions. This result implies the effects of other, stochastic factors, such as arthropod abundance or wildfires, although these cannot be directly tested with the available data. Our study illustrates that directional changes in average temperature and rainfall can affect the energetics and development of little brown bats. Ongoing nutritional stress associated with climate change may impact the viability of bat populations that are also threatened by white‐nose disease.

## Graphical abstract

Widespread declines in average forearm length of little brown bats ( *Myotis lucifugus* ) suggest landscape‐scale nutritional stress associated with shifting weather patterns.

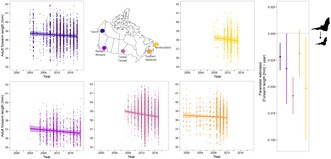

## 1 Introduction

Maintaining a positive energy balance is crucial for survival, yet physiological and environmental parameters challenge a species' ability to stave off energetic stress. At high latitudes, short active seasons limit time available to replenish energy stores, and winter brings long periods of low ambient temperatures and reduced access to food. Many species use prolonged, multi‐day to seasonal bouts of deep torpor (i.e., hibernation) to reduce metabolic rate and conserve energy during the winter (Geiser 2004; Wang and Wolowyk 1988). For hibernating species, overwinter survival and reproduction the following summer rely on adequate energy reserves stored during the preceding active season (Hranac et al. 2021; Humphries et al. 2003; Norquay and Willis 2014).

During the active season, heterothermic species can also use short, shallow bouts of daily torpor, lasting hours, to conserve energy during periods of low food availability (Körtner and Geiser 2000; Levy et al. 2011). As climate change increases the frequency and severity of inclement weather, which can limit foraging opportunities, daily torpor can allow energy conservation during periods of unpredictable resource availability (Geiser and Turbill 2009; Nowack et al. 2017). However, frequent use of daily torpor during gestation may jeopardize females' reproductive success by delaying parturition, while daily torpor during lactation may slow or limit post‐natal growth of offspring (Linton and Macdonald 2018). If females incur a greater fitness cost from using daily torpor, then the impacts of climate change on foraging opportunities may affect females more strongly than males.

Nutritional stress experienced by females prior to the breeding season can have intergenerational impacts through carry‐over effects, influencing the physiological condition and developmental trajectory of their offspring (Festa‐Bianchet 1998; Norris 2005). Poor nutritional conditions during pre‐natal and post‐natal development can result in reduced long‐bone length at maturity, and acute nutritional stress can cause reduced body mass (Hermanussen et al. 1996; Romano et al. 2010; Ellison 2003). Mounting evidence suggests that weather‐induced reductions in foraging opportunities during energetic bottlenecks (e.g., spring green‐up) may be driving reductions in body size across taxa (Aubry et al. 2013; Campbell et al. 2013; Canale et al. 2016; Cox et al. 2019; Holmes et al. 2021; Mason et al. 2014). Poor body condition in reproductive females can lead to a continuum of deteriorating fitness scenarios, from reduced offspring body size to decreased adult survival (Davy et al. 2022; Stapelfeldt et al. 2023).

However, the effects of climate‐induced nutritional stress vary among heterothermic, hibernating species (Stapelfeldt et al. 2023), due to variations in their life history strategies (Findlay‐Robinson et al. 2023; Wells et al. 2022). Climate change is also geographically, seasonally, and even diurnally asymmetrical (Cohen et al. 2012; Flato and Boer 2001; Cox et al. 2020), and weather variables can also interact or have seasonally and geographically variable impacts on animal physiology (Sheridan et al. 2022). As a result, the energetic impacts of climate change on heterothermic species are challenging to predict.

Body size in insectivorous bats varies spatially with primary productivity and temperature, which influence prey availability (Kelly et al. 2018; Alston et al. 2023). Declines in body size of little brown bats ( *Myotis lucifugus* ) over time would be consistent with expectations from chronic nutritional stress (Davy et al. 2022; Alston et al. 2023), and bats are more susceptible to energy deficits during some phenophases (i.e., phenological periods) than others because energetic costs and benefits of employing daily torpor vary throughout the active season (Balzer et al. 2022; Bergeson et al. 2021; Besler and Broders 2019; Davy et al. 2022; Dzal and Brigham 2012; Kurta et al. 1989). Rainfall and temperature can also have complex, interactive impacts on primary productivity, insect activity, foraging conditions, thirst, daily torpor arousal frequency and evaporative water loss, confounding the role of weather in energetic balance by bats (Adams 2010; Besler and Broders 2019; Jan et al. 2017; Sherwin et al. 2013). The complex interactive effects of phenophase, rainfall and temperature on energy balance by bats predict that the impacts of climate change on their body size may vary among regions. In this study, we sought to identify the relative effects of these factors on the body size of bats and quantify variation in these effects among regions.

**FIGURE 1:**
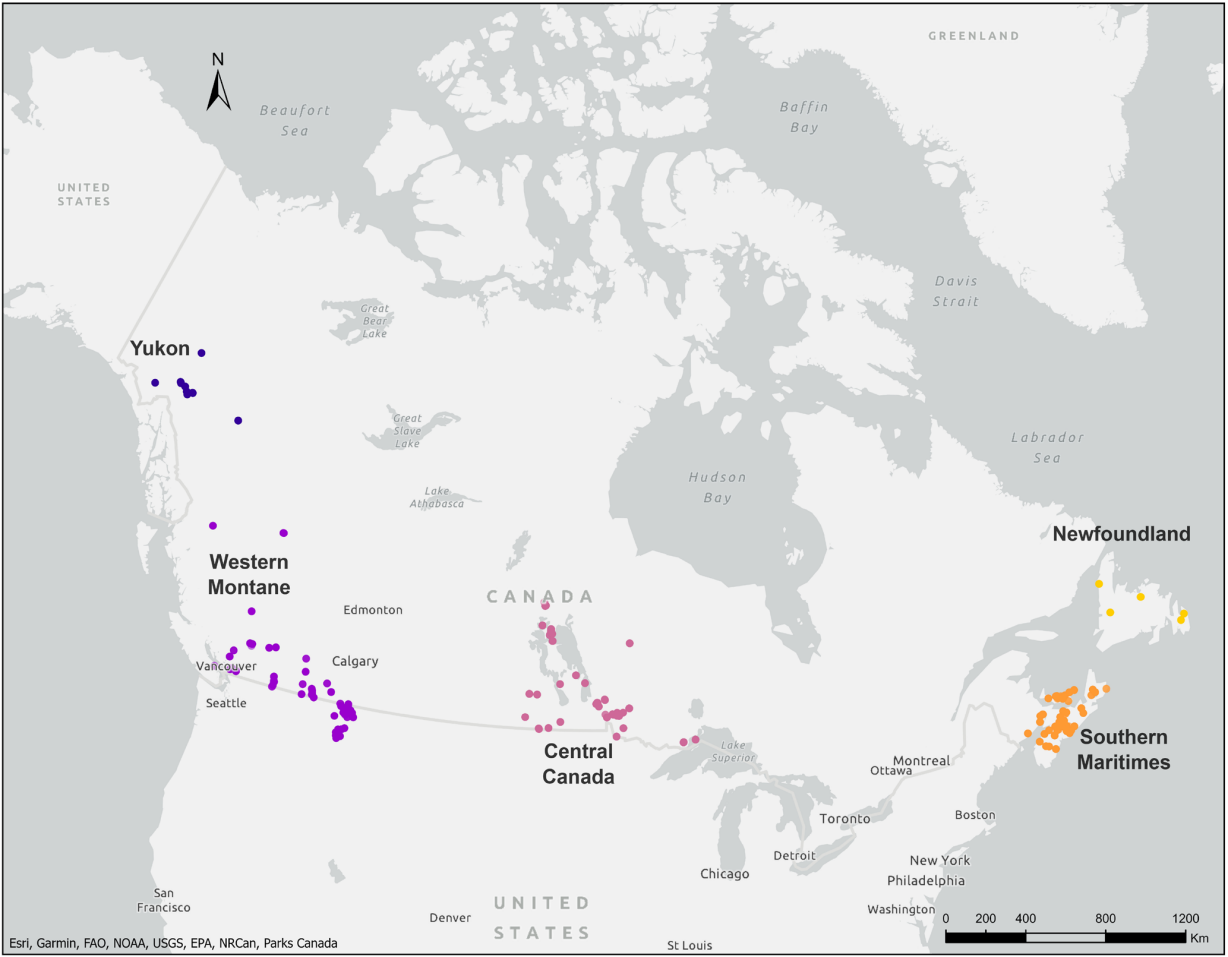
Little brown bat ( *Myotis lucifugus* ) capture sites coloured by study region, including Yukon (dark blue), Western Montane (purple), Central Canada (pink), Southern Maritimes (orange), and Newfoundland (yellow).

We measured spatiotemporal and phenological variation in body size of little brown bats, using multi‐year datasets collected in five regions spanning the northern part of their range (Figure 1). The objectives of our study were threefold: (1) to test whether reported local declines in average adult and juvenile forearm length over time were reflected at a larger spatial scale, indicating broad‐scale mechanisms such as climate change, (2) to test whether juvenile forearm length is influenced by the carry‐over effect of maternal foraging conditions before and after hibernation, predicting that juveniles would have shorter forearms following poor maternal foraging conditions (e.g., high rainfall), and (3) to test which factors best explained variation in body mass among phenophases and regions. For objective 3, we hypothesized that bats' ability to compensate for reduced foraging opportunities (e.g., by increased foraging effort or the use of daily torpor) was limited by the energetic demands of hibernation and reproduction. We predicted greater annual declines in body mass of bats captured during the most energetically constrained phenophases (e.g., following emergence from hibernation, or while females were nursing pups). We also predicted that local rainfall and temperature, which affect insect activity and bat energetics, would determine the magnitude of declines in body mass within each region.

## 2 Methods

### 2.1 Data Collection and Preparation

We leveraged morphometric data from little brown bats captured across Canada and northern Montana, USA (Figure 1). These data represented five geographically distinct study regions, with consistent data collection spanning a minimum of 8 years: (1) Yukon (2004–2018: 15 years), (2) Western Montane (2004–2019: 16 years), (3) Central Canada (2007–2017: 11 years), (4) Southern Maritimes (1999–2012: 14 years) and (5) Newfoundland (2008–2015: 8 years). The initial dataset included > 29,000 records. However, some of these data were collected from bats that had survived the arrival of bat white‐nose disease (WND). WND is associated with changes in average body mass (Cheng et al. 2019; Haase et al. 2021; Lacki et al. 2015), and possible effects of WND on forearm length have not been tested. To avoid the confounding effects of WND on bat body size, we excluded data collected during and after the year of confirmed invasion of WND in a region. We used invasion maps (Canadian Wildlife Health Cooperative 2025) to determine each region's year of confirmed WND invasion. We retained 21,840 records for further analysis.

Capture and handling of bats complied with guidelines from the American Society of Mammalogists (Sikes 2016) and was permitted by the relevant provincial or state wildlife management agency in each case. Bats were captured between late April and mid‐October for previous studies using mist nets or harp traps, either at emergence from roosts in trees, buildings, or bat boxes, by mist‐netting in foraging habitat, or by trapping at the entrances to caves or abandoned mines used for swarming. Phalangeal joint ossification was assessed to differentiate adults (> 1 year old) from young of the year that survived to volancy (juveniles; Kunz and Anthony 1982). Sex was determined by examination of the external genitalia. In some regions, pregnancy was assessed by gently palpating the abdomen of adult females (Kunz and Anthony 1982), meaning that late‐stage pregnancies (i.e., increased body mass due to pregnancy) were accounted for in the dataset, although early‐stage pregnancies would be undetectable. The forearm length of each bat was measured with digital callipers (±0.1 mm). Bats were individually marked with lipped aluminium bands (Porzana, U.K.) on the forearm, or with passive integrated transponder (PIT) tags (Trovan Electronic Identification Systems, UK) inserted subcutaneously between the scapulae and for individuals that were subsequently recaptured, we used the forearm measurement from the first capture in our analyses. Each bat was also weighed using a digital scale (±0.1 g). We considered markers of access to nutrition at two temporal scales: forearm length provided a proxy for relative access to nutrition during development, and mass provided a proxy for energy reserves at the time of capture (Davy et al. 2022).

**FIGURE 2:**
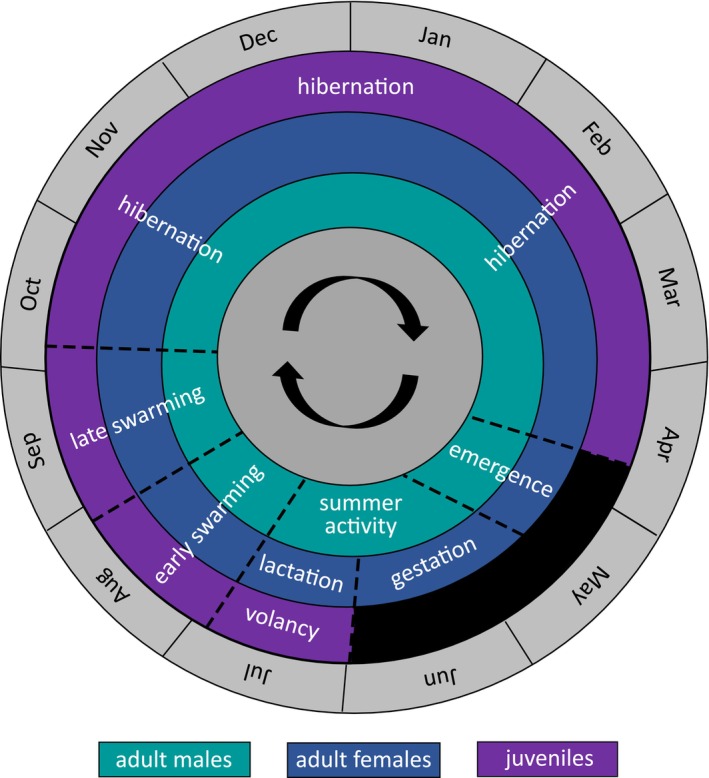
Phenology wheel illustrating the annual life cycle events of the little brown bat ( *Myotis lucifugus* ) in the northern part of its range. The wheel is divided into 12 segments, each representing a month of the year. Key phenophases (i.e., phenological periods) such as emergence, gestation and swarming are depicted in their respective months and coloured by demographic group. The active season includes every phenophase except for hibernation.

We defined seven phenophases of the active season (Table 1, Figure 2): emergence (from hibernation), gestation (adult females), lactation (adult females), summer activity (adult males), volancy (juveniles), early swarming and late swarming. We defined the time period of each phenophase using prior knowledge of bat behaviour (Figure 2), and the inflection points of smooth fitted generalized additive models of body mass and ordinal day (fitted with the ‘mgcv’ package; v1.8.42; Wood 2011) for each study region and demographic group (adult female, adult male, and juvenile). Inflection points were identified using the ‘gratia’ package (v0.8.1; Simpson 2023) to calculate derivatives of the smooth estimates of ordinal day. Phenophase duration and shape of the smoothed fit varied by demographic group and study region (Figure S1). Bats were not captured at all study regions during all phenophases in each year.

**TABLE 1:** Phenophases of the active season of temperate, hibernating bats.

Phenophase	Approximate timing	Bat activity	Demographic group
Emergence	Late April to mid‐May	Emerging from hibernation; provisioning for summer activity (males and non‐reproductive females) and reproduction (reproductive females)	Adult females and adult males
Gestation	Mid‐May to late June	Provisioning of fetus	Adult females
Summer activity	Mid‐May to late July	Foraging; preparation for mating	Adult males
Lactation	July	Pup provisioning	Adult females
Volancy	July	Pups begin flight and are weaned as they learn to forage independently	Juvenile females and juvenile males
Early swarming	Late July to late August	Mating and dispersal	Adult females, adult males, juvenile females and juvenile males
Late swarming	Late August to early October	Mating and preparation for hibernation	Adult females, adult males, juvenile females and juvenile males

To examine the interactive effects of temperature and rainfall (focal variables) on body size during the active season we used the ‘weathercan’ (v0.6.2; LaZerte and Albers 2018) and ‘rnoaa’ (v1.3.8; Chamberlain and Hocking 2023) packages to obtain weather data. We extracted daily rainfall volume and daily average minimum temperature from weather stations nearest to each capture site, with all weather stations being maintained by the federal government. The average distance from weather stations to capture sites was 35 km (range = 0.7–198 km). The weather stations recorded rainfall as a daily total. For each individual bat, we defined a pre‐capture period between ordinal day 107 [~17 April] for adults (Slough and Jung 2008; Norquay and Willis 2014) or earliest volancy for juveniles, and the day preceding capture. For each year and capture site, we calculated the proportion of rain days (days with > 1 mm rain; PropRain) and the proportion of warmer‐than‐average days (days above the study period average; PropWarm) in the pre‐capture period. Statistically significant weather interactions (*p* < 0.05; PropRain × PropWarm) were retained in the models, otherwise the main effects were retained separately. The simple slopes of significant weather interactions were examined using the ‘interactions’ package (v1.1.5; Long 2019).

### 2.2 Modelling Weather Trends Over the Study Period

To describe changes in weather over the study period, we ran linear mixed models (LMM) using the ‘lme4’ package (Bates et al. 2015) for each phenophase and region. Models used mean minimum temperature or the total number of rain days (TotalRain) as the response variable, year as a fixed effect, and weather station as a random effect.

### 2.3 Modelling Forearm Length Over the Study Period

We ran LMMs to test for changes in average forearm length of bats over time. Using a separate model for each region, we tested how adult forearm length (response variable) was predicted by year (focal fixed effect), including sex as a fixed effect because little brown bats exhibit sexual size dimorphism (Williams and Findley 1979). We included capture site as a random effect, with the exception of models with a small number of sites (< 5; Gelman and Hill 2007).

We ran similar models to quantify temporal trends in juvenile forearm length in each region. Juvenile bats' forearms grow substantially for ~21 days after birth (Baptista et al. 2000; Kunz and Anthony 1982), after which growth slows. To ensure our results were not affected by continued, substantial growth in forearm length after volancy and potential variation in capture dates among years, we used five linear models to test whether ordinal date predicted juvenile forearm length in each region. We found no effect of ordinal date on forearm length of captured juveniles, so we proceeded with analyses. Models with juvenile forearm length (response variable) included year, sex and several weather variables (fixed effects). As described below, the weather variables also allowed us to test for carry‐over effects on juvenile forearm length.

### 2.4 Modelling Carry‐Over Effects on Juvenile Forearm Length

Unlike data collected from adult bats with unknown birth years, juvenile forearm lengths could be associated with the year in which juveniles were born, so juvenile growth could be related to foraging conditions experienced by their mothers in the year preceding birth. We tested our carry‐over hypothesis at two temporal scales. First, we predicted that poor weather conditions during emergence could limit a female's capacity to provision her developing fetus. Our first model of juvenile forearm length (response variable) included fixed effects of year and sex, as detailed above. It also included the main effects and interaction between the total number of rain days (TotalRain) and the total number of warmer‐than‐average days (TotalWarm) during the emergence phenophase preceding the birth of juveniles at their birth site, in their birth‐year. Second, we predicted that mothers experiencing such conditions in the previous year's active season would also produce pups with shorter forearms. We ran a second set of models like the first but using the TotalRain and TotalWarm from the active season in the year preceding the juveniles' birth year. Statistically significant weather interaction terms (*p* < 0.05; TotalRain × TotalWarm) were retained in the models; otherwise, the main effects were retained separately.

To more directly test the association between juvenile forearm length and females' access to food before and after hibernation, we searched the data for cases where capture data were available for juveniles at a site, and from adult females captured at that site prior to those juveniles' birth. We built separate LMMs to test whether juvenile forearm length (response variable) in each year was predicted by mean adult female body condition index (BCI; defined as mass/forearm [Pearce et al. 2008]) in the late swarming phenophase (‘previous active season’), or in the spring emergence period preceding the birth of each juvenile cohort. We used BCI as the focal predictor to account for the allometric relationship we found between adult body mass and adult forearm length (Table S1).

### 2.5 Modelling Predictors of Body Mass Across Phenophases and Regions

Bat body mass reflects recent energy reserves, and we next quantified variation in energetic stress among phenophases in the active season. We ran LMMs to test how adult and juvenile body mass were predicted by year and weather. We modelled adult body mass (response variable) for each geographic region, sex and phenophase, including fixed effects of year, PropRain and PropWarm (focal predictor variables). To account for other factors affecting body mass, we included the fixed effects of forearm length and ordinal day as a 2nd order polynomial term to account for non‐linear relationships (Davy et al. 2022). We included the reproductive status of females captured during the mid‐late spring (pregnant or not pregnant, where such data were available). Models of juvenile body mass (response variable) for each region also included fixed effects of year, PropRain, PropWarm, forearm length, sex and polynomial ordinal day. Each body mass model included the interaction between PropRain and PropWarm (PropRain × PropWarm) to test whether the effects of rainfall on body mass were mediated by temperature. Finally, body mass models included capture site as a random effect, with the exception of models with a small number of sites (< 5; Gelman and Hill 2007; Tables S1–S3). Statistically significant weather interactions (*p* < 0.05; PropRain × PropWarm) were retained in the models; otherwise, the main effects were retained separately.

Prior to modelling, body mass data were natural log‐transformed to account for a skewed distribution. Assumptions of model fit were checked with diagnostic plots of residuals using the ‘performance’ package (v0.10.3; Lüdecke et al. 2021). We retained only the models with ≥ 10 observations per term except for one model with a predicted *R* ^2^ that was within 0.04 of the adjusted *R* ^2^, indicating no issues with overfitting (Allen 1971). We calculated the marginal semi‐partial *R* ^2^ attributed to each model covariate using the ‘part*R* ^2^’ package (Stoffel et al. 2021). When plotting select model results, we standardized continuous predictor variables (mean = 0, SD = 1) to facilitate effect size comparison across predictor variables and models. All analyses were performed using R Statistical Software (v4.2.3; R Core Team 2021).

## 3 Results

### 3.1 Weather Trends Over the Study Period

Mean minimum temperature remained stable over the study period in some regions and increased in others, with a varying magnitude of effect of year on temperature among phenophases (Table S4a, Figure S3). The greatest estimated increase was 0.135°C per year during gestation in Central Canada (1.485°C increase during gestation over the study period). The trend in total rain days varied among phenophases (Table S4b, Figure S3). Most regions saw fewer rain days over the study period during emergence and/or gestation, with the strongest drying trends occurring in Newfoundland (0.629 fewer rain days per year during emergence and 0.857 fewer rain days during gestation). In general, there was little change in the number of rain days during lactation, and variability among regions in the early and late swarming periods, although the Yukon showed a relatively strong drying trend in late swarming. The greatest increase in the number of rain days per year occurred in the Southern Maritimes region with an additional 0.224 days during early swarming and 0.292 during late swarming (approximately 3.136 and 4.088 additional rain days over the study period, respectively).

### 3.2 Forearm Length Over the Study Period

**FIGURE 3:**
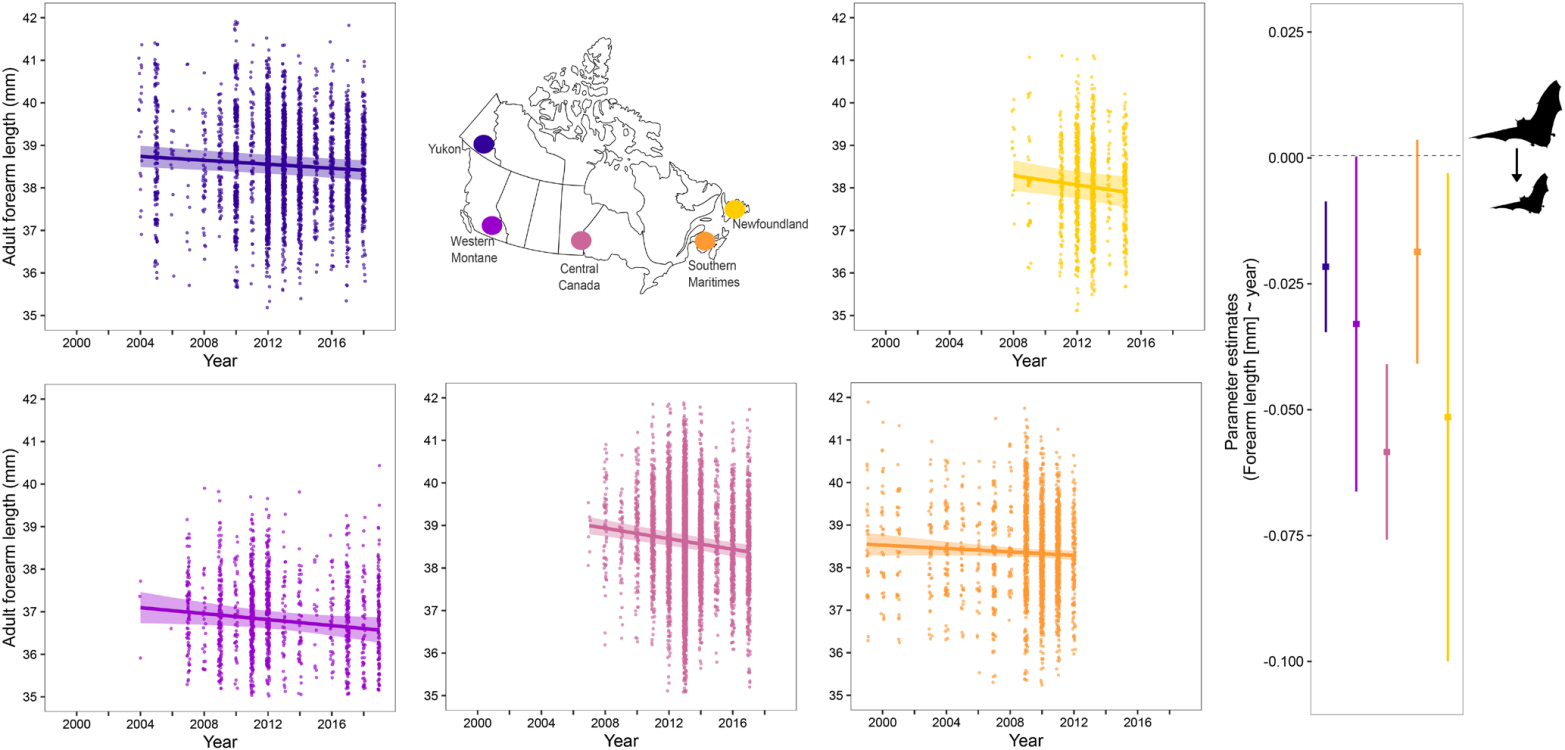
Model fits (with shaded 95% confidence intervals) and parameter estimate points (with 95% confidence interval bars) from linear mixed‐effect models examining the change in forearm length of adult little brown bat ( *Myotis lucifugus* ) over the respective study periods. Parameter estimates below zero indicate a decrease in average bat forearm length over the study period.

The dataset included forearm length measurements from 19,056 unique, marked individuals (10,146 adult females, 5311 adult males, 1788 juvenile females and 1811 juvenile males). Adult forearm length decreased, on average, in all five regions over their respective study periods (Table 2, Figure 3). The magnitude of the estimated decrease varied among regions, ranging from 0.023 to 0.062 mm/year for adults in the Yukon and Central Canada, respectively. For an adult bat in Central Canada, this represented an estimated decrease in forearm length of 1.8%, on average, over an 11‐year study period. Juvenile forearm length also decreased, on average, in all five regions over their respective study periods (Table 2a). The magnitude of the estimated decrease varied among regions, ranging from 0.037 mm/year for juveniles in the Yukon and Central Canada to 0.354 mm/year for juveniles in Newfoundland.

**TABLE 2:** Parameter estimates (*β*), 95% confidence intervals (CI) and marginal semi‐partial *R* ^2^ (italics) from linear mixed‐effects models examining the effects of year, temperature and rainfall on forearm length (mm) of adult and juvenile little brown bats ( *Myotis lucifugus* ) across five geographic regions.

Adults	Yukon			Western Montane			Central Canada			Southern Maritimes			Newfoundland		
	*n* obs. = 3316 sites = 11			*n* obs. = 1671 sites = 48			*n* obs. = 6778 sites = 31			*n* obs. = 2581 sites = 44			*n* obs. = 1111 sites = 5		
	*β*	CI	*R* ^2^	*β*	CI	*R* ^2^	*β*	CI	*R* ^2^	*β*	CI	*R* ^2^	*β*	CI	*R* ^2^
Year^a^	**−0.023**	**−0.037, −0.010**	*0.004*	**−0.035**	**−0.070, −0.000**	*0.000*	**−0.062**	**−0.080, −0.044**	*0.007*	−0.020	−0.043, 0.003	*0.002*	**−0.055**	**−0.105, −0.004**	*0.005*
Sex (male)	—	—	—	**−0.636**	**−0.781, −0.490**	*0.040*	**−0.366**	**−0.423, −0.310**	*0.023*	**−0.431**	**−0.525, −0.337**	*0.036*	—	—	—

(a) Juveniles	*n* obs. = 765 sites = 10			*n* obs. = 198 sites = 30			*n* obs. = 1858 sites = 29			*n* obs. = 634 sites = 34			*n* obs. = 389 sites = 4		
No weather	*β*	CI	*R* ^2^	*β*	CI	*R* ^2^	*β*	CI	*R* ^2^	*β*	CI	*R* ^2^	*β*	CI	*R* ^2^
Year^a^	**−0.037**	**−0.067, −0.007**	*0.010*	**−0.149**	**−0.268, −0.031**	*0.118*	**−0.037**	**−0.063, −0.011**	*0.005*	**−0.071**	**−0.110, −0.032**	*0.060*	**−0.354**	**−0.447, −0.260**	*0.124*
Sex (male)	**−0.399**	**−0.564, −0.234**	*0.027*	**−0.871**	**−1.210, −0.532**	*0.087*	**−0.261**	**−0.359, −0.164**	*0.013*	**−0.460**	**−0.638, −0.281**	*0.032*	**−0.611**	**−0.824, −0.399**	*0.077*

(b) Juveniles	*n* obs. = 749 sites = 10			*n* obs. = 191 sites = 28			*n* obs. = 1636 sites = 27			*n* obs. = 634 sites = 34			*n* obs. = 389 sites = 4		
Emergence weather	*β*	CI	*R* ^2^	*β*	CI	*R* ^2^	*β*	CI	*R* ^2^	*β*	CI	*R* ^2^	*β*	CI	*R* ^2^
TotalRain^a^	0.036	−0.033, 0.105	*0.002*	−0.076	−0.204, 0.051	*0.026*	—	—	—	−0.026	−0.073, 0.020	*0.000*	—	—	—
TotalWarm^a^	**−0.039**	**−0.078, −0.000**	*0.003*	0.024	−0.059, 0.106	*0.015*	—	—	—	−0.030	−0.068, 0.007	*0.012*	—	—	—
TotalRain × TotalWarm^a^	NS	NS	NS	NS	NS	NS	**−0.033**	**−0.048, −0.017**	*0.016*	NS	NS	NS	**−0.017**	**−0.028, −0.006**	*0.023*
− 1 SD	—	—	—	—	—	—	**0.090**	**0.033, 0.146**	—	—	—	—	**0.089**	**0.047, 0.131**	—
Mean	—	—	—	—	—	—	0.003	−0.029, 0.036	—	—	—	—	**0.050**	**0.018, 0.082**	—
+ 1 SD	—	—	—	—	—	—	**−0.083**	**−0.132, −0.034**	—	—	—	—	0.011	−0.029, 0.051	—
Year^a^	**−0.063**	**−0.113, −0.014**	*0.006*	**−0.174**	**−0.303, −0.045**	*0.136*	−0.011	−0.050, 0.029	*0.000*	**−0.080**	**−0.127, −0.034**	*0.059*	−0.078	−0.224, 0.068	*0.003*
Sex (male)	**−0.402**	**−0.567, −0.236**	*0.027*	**−0.873**	**−1.222, −0.524**	*0.080*	**−0.255**	**−0.359, −0.151**	*0.013*	**−0.454**	**−0.632, −0.275**	*0.031*	**−0.595**	**−0.797, −0.393**	*0.080*

(c) Juveniles	*n* obs. = 749 sites = 10			*n* obs. = 191 sites = 28			*n* obs. = 1636 sites = 27			*n* obs. = 567 sites = 34			*n* obs. = 389 sites = 4		
Previous year weather	*β*	CI	*R* ^2^	*β*	CI	*R* ^2^	*β*	CI	*R* ^2^	*β*	CI	*R* ^2^	*β*	CI	*R* ^2^
TotalRain	—	—	—	−0.023	−0.056, 0.010	*0.034*	—	—	—	0.007	−0.000, 0.015	*0.012*	—	—	—
TotalWarm	—	—	—	−0.001	−0.034, 0.032	*0.000*	—	—	—	0.005	−0.005, 0.014	*0.000*	—	—	—
TotalRain × TotalWarm^a^	**0.002**	**0.001, 0.003**	*0.017*	NS	NS	NS	**−0.002**	**−0.003, −0.001**	*0.008*	NS	NS	NS	**−0.001**	**−0.002, −0.000**	*0.017*
− 1 SD	**−0.049**	**−0.075, −0.023**	—	—	—	—	**0.036**	**0.010, 0.063**	—	—	—	—	0.015	−0.007, 0.037	—
Mean	−0.007	−0.026, 0.012	—	—	—	—	0.007	−0.004, 0.019	—	—	—	—	−0.005	−0.024, 0.013	—
+ 1 SD	**0.035**	**0.000, 0.070**	—	—	—	—	**−0.022**	**−0.037, −0.006**	—	—	—	—	**−0.026**	**−0.051, −0.000**	—
Year^a^	−0.017	−0.074, 0.040	*0.002*	**−0.213**	**−0.360, −0.067**	*0.158*	−0.014	−0.053, 0.024	*0.000*	−0.032	−0.075, 0.011	*0.011*	**−0.275**	**−0.454, −0.095**	*0.023*
Sex (male)	**−0.409**	**−0.575, −0.244**	*0.024*	**−0.876**	**−1.220, −0.533**	*0.090*	**−0.254**	**−0.359, −0.150**	*0.012*	**−0.459**	**−0.647, −0.271**	*0.038*	**−0.604**	**−0.813, −0.396**	*0.078*

*Note:* We present three juvenile forearm model sets; one that includes only year and sex as fixed effects (a), one that includes year, sex, and weather conditions in the emergence phenophase prior to birth (b) and one that includes year, sex, and weather conditions in the previous year during the active season (c). Bold text indicates variables with confidence intervals not overlapping zero.

Abbreviations: dash, not applicable; NS, not significant (*p* ≥ 0.05); obs., observations; TotalRain, total number of rain days during the emergence phenophase prior to birth of juveniles; TotalRain × TotalWarm, the interactive effect of TotalRain at three levels of TotalWarm (−1 SD, Mean and +1 SD); TotalWarm, total number of warmer‐than‐average days during the emergence phenophase prior to birth of juveniles (please refer to Section 2 for details).

^a^ Focal variables.

### 3.3 Carry‐Over Effects on Juvenile Forearm Length

**FIGURE 4:**
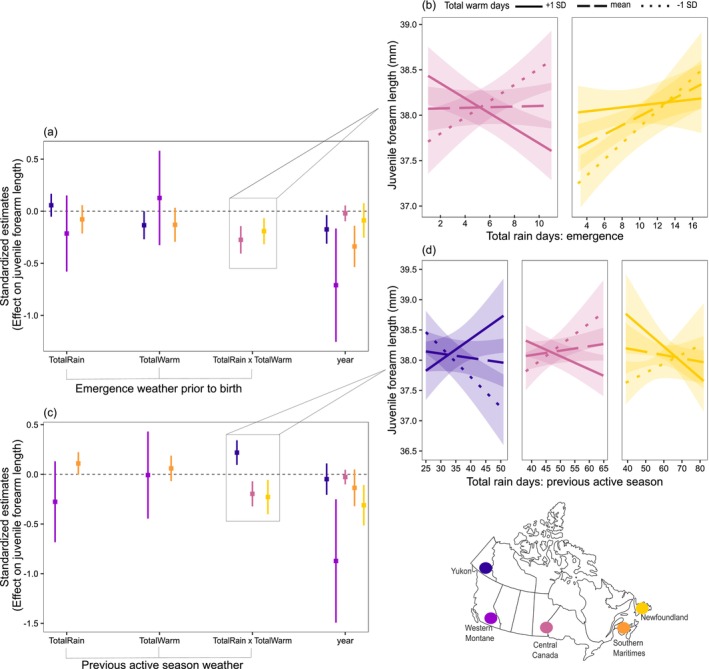
Standardized parameter estimates (with 95% confidence interval bars) and model fits (with shaded 95% confidence intervals) from linear mixed‐effects models predicting weather and temporal effects on juvenile forearm length (mm) of little brown bat ( *Myotis lucifugus* ). Predictors of juvenile forearm length included (a, c) year, (a, b) weather experienced by adult females in the emergence phenophase prior to birth of juveniles, and (c, d) weather experienced by adult females during the active season in the year preceding birth of juveniles. Interaction plots (b, d) show the effect of TotalRain on juvenile forearm length at three levels of TotalWarm (−1 SD, Mean, +1 SD).

When weather was incorporated into models of juvenile forearm length, temperature and rainfall experienced by reproductive females before and after hibernation explained some proportion of the variation in juvenile forearm length in 3 of 5 study regions (Table 2b,c, Figure 4). The strongest weather‐based predictor of juvenile forearm length was the interaction between TotalRain and TotalWarm. For example, in Central Canada, juvenile forearm length decreased by 0.083 mm for each rain day in the emergence phenophase prior to birth in years with more warmer‐than‐average days (+1 SD) during this period. In cooler years with fewer warmer‐than‐average days (−1 SD) during emergence, juvenile forearm length increased by 0.090 mm with each additional rain day (Table 2b, Figure 4b). Another noteworthy weather‐based predictor of juvenile forearm length was warmer‐than‐average weather during emergence in the Yukon; juvenile forearms decreased by 0.039 mm for each warmer‐than‐average day experienced by females during emergence (Table 2b, Figure 4a).

**FIGURE 5:**
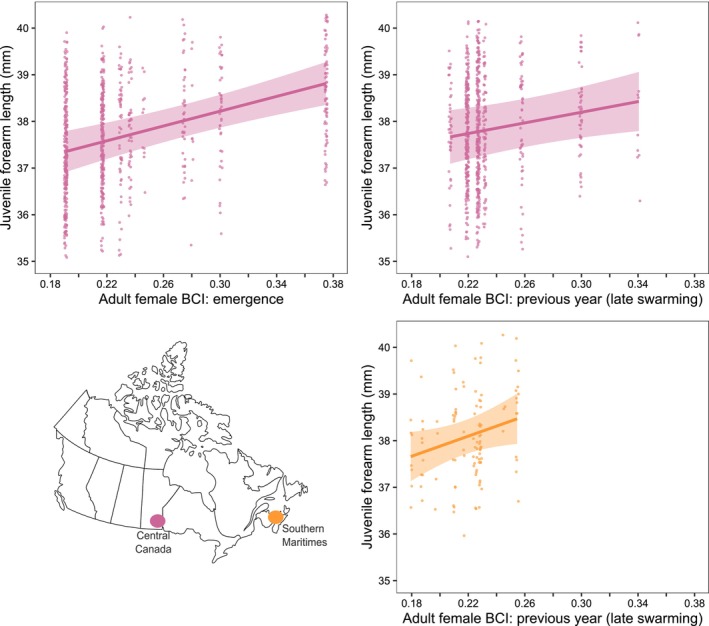
Model fits (with shaded 95% confidence intervals) from linear mixed‐effects models predicting effects of adult female body condition index (BCI) in emergence and the previous year's late swarming period on juvenile forearm length (mm) of little brown bat ( *Myotis lucifugus* ).

Only data from Central Canada and Southern Maritimes included captures of juveniles and a matching cohort of adult females from the same or preceding year. Results from these regions suggest that the BCI of adult females captured in late swarming explained a proportion of the variation in juvenile forearm length the following year (*β* = 5.695 to 10.757, semi‐partial *R* ^2^ = 0.022–0.086; Table 3, Figure 5). In Central Canada, the BCI of adult females captured in emergence also explained a proportion of the variation in juvenile forearm length (*β* = 7.926, semi‐partial *R* ^2^ = 0.105).

**TABLE 3:** Parameter estimates (*β*), 95% confidence intervals (CI), and marginal semi‐partial *R* ^2^ (italics) from linear mixed‐effects models examining the effect of average adult female body condition index (BCI) in emergence and in the previous year's late swarming period on forearm length (mm) in juvenile little brown bats ( *Myotis lucifugus* ).

	Central Canada (emergence)			Central Canada (previous year late swarming)			Southern Maritimes (previous year late swarming)		
	*n* obs. = 781 sites = 6			*n* obs. = 731 sites = 5			*n* obs. = 119 sites = 7		
	*β*	CI	*R* ^2^	*β*	CI	*R* ^2^	*β*	CI	*R* ^2^
Adult female BCI (emergence)^a^	**7.926**	**4.794, 11.058**	*0.105*	—	—	—	—	—	—
Adult female BCI (previous year late swarming)^a^	—	—	—	**5.695**	**1.073, 10.318**	*0.022*	**10.757**	**0.545, 20.968**	*0.086*
Year	−0.011	−0.143, 0.121	*0.000*	**−0.301**	**−0.468, −0.134**	*0.031*	**−0.543**	**−0.832, −0.253**	*0.144*
Sex (male)	**−0.284**	**−0.440, −0.129**	*0.014*	**−0.328**	**−0.490, −0.166**	*0.016*	**−0.425**	**−0.777, −0.074**	*0.030*

*Note:* Adult female BCI (emergence) is calculated by dividing mass by forearm for adult females captured in the emergence phenophase prior to the birth of a matching cohort of juveniles by site and year. Adult female BCI (previous year late swarming) is the same calculation but for adult females caught during the late swarming phenophase of the previous year (please refer to Section 2 for details). Bold text indicates variables with confidence intervals not overlapping zero.

Abbreviations: dash, not applicable; obs, observations.

^a^ Focal variables.

### 3.4 Predictors of Body Mass Across Phenophases and Regions

**FIGURE 6:**
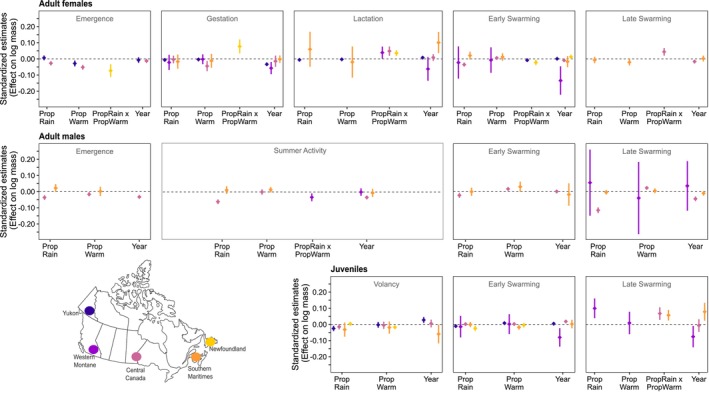
Standardized parameter estimates (dots) and 95% confidence intervals (bars) for linear mixed‐effect models predicting the effects of proportion of rain days (‘PropRain’), proportion of warmer‐than‐average days (‘PropWarm’), their interaction (‘PropRain × PropWarm’), and year on log body mass (g) of adult male, adult female, and juvenile little brown bats ( *Myotis lucifugus* ) among phenophases. Confidence intervals are smaller than the point size when not visible. Parameter estimates above zero indicate an increase in bat body mass with the predictor variable and estimates below zero indicate a decrease. Interactions between PropRain and PropWarm can be further examined in Figure S2 to aid interpretation of the direction of effect at different levels of the moderator (PropWarm).

The final data set used to quantify variation in body mass among phenophases and regions included measures of 21,840 marked individuals (13,009 adult females, 5108 adult males, 1851 juvenile females and 1872 juvenile males). The effects of temperature and rainfall had varied impacts on body mass across phenophases, demographic groups, and regions (Tables S1–S3, Figure 6). The influence of PropRain as a main effect was mostly negative, with the greatest impact on adult bats in Central Canada during late swarming. For example, body mass in late swarming decreased by 2% for every 1% increase in PropRain for adult males, and between 1% and 2% for adult females, depending on how well warmer‐than‐average temperatures mediated the decrease in mass (Tables S1 and S2, Figures 6 and S2). For some regions, the effect of PropWarm on body mass was most negative in the early part of the active season (emergence and gestation), becoming increasingly positive as the active season progressed. For example, adult female body mass in the Yukon and Central Canada decreased by 0.3% and 0.7%, respectively, for each 1% increase in PropWarm during the emergence phenophase, whereas this decrease declined towards the end of the active season (Table S1, Figure 6).

The significance and direction of the interaction between PropRain and PropWarm varied across demographic groups, regions, and phenophases (Tables S1–S3, Figures 6 and S2). For example, the effect of PropRain on adult female body mass in Central Canada was mediated by increased PropWarm during lactation and late swarming phenophases, yet this interaction did not predict adult male body mass in Central Canada in any phenophase. For adult females in Newfoundland, body mass was affected by PropRain × PropWarm in all sampled phenophases, but the direction of the interaction showed opposing patterns between the middle of the active season and the periphery (Table S1; Figures 6 and S2). For example, the effect of PropRain on body mass was negatively mediated by increased PropWarm during emergence, positively mediated during gestation and lactation, and negatively mediated during early swarming.

The magnitude of temporal declines in body mass also varied across phenophases, demographic groups and regions, with the greatest reductions occurring in adult female and juvenile Western Montane bats captured during early and late swarming (Tables S1–S3, Figure 6). On average, body mass of adult female and juvenile Western Montane bats caught during early swarming declined by 3.5% and 2.5% each year, respectively. Adult body mass of bats in Central Canada also decreased over the study period in phenophases on the periphery of the active season. For example, body mass of adult females and adult males decreased annually on average by 1% during emergence and 3% during late swarming, while the decrease was less pronounced during gestation, lactation and early swarming. In datasets where pregnancy was assessed, heavily pregnant bats (i.e., detectable pregnancies) weighed 0.141 g more, on average, than adult females that were not obviously pregnant (Table S1).

## 4 Discussion

Our analyses revealed a widespread decrease in forearm length of a broadly distributed aerial insectivore, the little brown bat. Our results were generally consistent with predictions of shifting weather patterns associated with climate change as a factor in these declines, and identified phenophases during which a high latitude hibernating bat was more vulnerable to nutritional stress. We detected declines in the average forearm length of little brown bats in each of our study regions, supporting the contention that landscape‐scale nutritional stress has impacted little brown bats across the northern part of their range, generalizing earlier findings from the Yukon (Davy et al. 2022). Those declines may reflect changes in insect abundance. While tracking insect abundance over time is challenging and robust data do not exist at the scale of our study, growing consensus supports insect declines in some regions (Scherber et al. 2026). Our results could also be explained by weather‐related declines in foraging opportunities, which are supported by our analyses, or a combination of both mechanisms.

As predicted, we found that mothers' access to nutrition before and after hibernation exerts a carry‐over effect on the forearm length of juveniles, and we observed some consistent effects of weather on body size within age‐sex classes and phenophases. In a previous study, body mass of little brown bats was greater in years when spring and autumn temperatures and net primary productivity (NPP) were high, yet overall, body mass decreased over time despite increases in temperature and NPP (Alston et al. 2023). Our results may resolve this apparent contradiction, as we found that body mass was affected differently by temperature, rainfall and the interaction between these factors, depending on the phenophase.

Overall, our findings contribute to a mechanistic understanding of climate‐related energetic stress in bats and illustrate the inherent challenges of studying a case of widespread, apparent nutritional stress in a system where data on prey abundance are essentially non‐existent. Weather interactions and the energy reserves of adult females during late swarming and emergence were associated with carry‐over effects on juvenile body size in the subsequent summer. Bats at high latitudes must store a considerable amount of fat in the pre‐hibernation period, which coincides with the energetic constraints of swarming activity and increasingly wet and cold conditions. Pre‐hibernation body condition is a predictor of post‐hibernation body condition (Hranac et al. 2021; Kunz et al. 1998; Norquay and Willis 2014) which in turn determines the remaining energy available to survive the winter and spring (Hranac et al. 2021), fight disease (Haase et al. 2021), reproduce (Jonasson and Willis 2011) and provision offspring (this study). For hibernating species that produce a maximum of one offspring per year, the fitness costs of poor pre‐hibernation body condition are exceptionally high. In Central Canada, body mass declined up to 9.5 times faster after rainy late swarming weather than after rainy emergence weather. When lighter adult females were observed in late swarming and emergence, juveniles born in the subsequent summer had shorter forearms, on average. Climate change projections for the next 50 years predict a continent‐wide increase in autumn rainfall, with an estimated increase of 15% predicted for some regions (Prairie Climate Centre 2019).

Other ongoing changes in bat body size associated with regional weather trends highlighted how global changes in climate can produce diverse outcomes among regions. One might expect that bats would be heavier following warmer‐than‐average weather due to the reduced cost of thermoregulation and the increased availability of insects (Anthony et al. 1981; Currie et al. 2015). However, we found that little brown bats emerging from hibernation had lower body mass after an increase in warmer‐than‐average weather in the Yukon, and after an increase in warmer‐than‐average, wetter weather in Central Canada and Newfoundland. During the emergence phenophase, temperate bats rely heavily on daily torpor to cope with unpredictable weather and food supply (Linton and Macdonald 2018; Zahn et al. 2007). Bats use cold ambient temperature as a cue to initiate daily torpor, conserving energy while insect prey are scarce (Wojciechowski et al. 2007). Little brown bats in poorer condition select cooler microclimates in which to intensify their torpid state and maximize energy conservation (Boyles et al. 2007). Conversely, warm ambient temperatures typically imply availability of insect prey, and bats likely respond with more frequent arousal, and longer bouts of activity (Fjelldal et al. 2021; Park et al. 2000; Stawski and Geiser 2012). An increase in the number of warmer‐than‐average days during emergence may prompt bats to become active earlier, before insect abundance increases (Meyer et al. 2016), creating a phenological mismatch. When increased rainfall further limits foraging opportunities, bats experience reduced body condition and reproductive output (Grindal et al. 1992; Linton and Macdonald 2018; Lučan et al. 2013). Climate change projections under high carbon emission scenarios predict warmer and wetter springs in the near future, ranging from 1.4°C to 2.2°C warmer, and 4% to 12% wetter in the next 50 years (Prairie Climate Centre 2019).

In Central Canada and Newfoundland, signs of energetic stress were also associated with a different interaction between temperature and rainfall during emergence. Weather during emergence in these regions was drier but not warmer over the study period, and this trend was also associated with decreased body mass. Dry conditions combined with high rates of evaporative water loss in little brown bats (McGuire et al. 2021) might also increase the frequency of arousing from daily torpor (Ben‐Hamo et al. 2013; Thomas and Cloutier 1992). If temperatures during emergence are cool enough to require the use of daily torpor or inhibit insect activity, yet bats are arousing due to dehydration, excessive energy would still be expended during a critical time for survival and reproduction.

Consistent with our predictions, increased rainfall had the greatest impact during energy bottlenecks like emergence and late swarming, but we also predicted that bats might be equally vulnerable while lactating (Kurta et al. 1989). In contrast, we found that over the study period, adult females captured during the lactation period were less affected by changing weather patterns than bats in emergence and swarming, presumably because the weather trends were more favourable relative to their impacts on bat mass. Females captured during the lactation period were still significantly lighter after cooler, wetter weather, but Western Montane was the only region that experienced a strong cooling trend during the lactation period. If thermoregulatory capacity is not exceeded, warm temperatures during lactation may provide benefits to lactating females and developing juveniles (Lausen and Barclay 2006; Noakes et al. 2021). However, we found that bats captured during lactation were also lighter after warmer, drier weather. Lactating females require more water than non‐reproductive females (Adams and Hayes 2008), and more frequent and further trips to drink during dry weather may quickly drain energetic resources, thus slowing juvenile development (Tuttle 1976). In the southern Rocky Mountains, an unusually warm, dry period reduced bat reproductive output (Adams 2010), further implying that higher rates of evaporative water loss can negatively affect body condition and ultimately fitness. Compared to spring and fall, summer climate change projections for rainfall over the next 50 years are more geographically variable, indicating more localized impacts during the typical lactation period (~July). Under high carbon emission scenarios, July is predicted to get 2%–9% wetter in the Yukon, Southern Maritimes, and Newfoundland, while the Western Montane varies from 9% drier to 2% wetter, and Central Canada varies from 3% drier to 4% wetter (Prairie Climate Centre 2019). A July warming trend from 1.6°C to 2.1°C is predicted continent‐wide (Prairie Climate Centre 2019).

Despite the weather variables associated with changing body size in little brown bats, we were unable to explain the remaining variance in body size trends, indicating the relative strength of other, unmeasured covariates in our study systems. For example, there were no significant weather predictors of juvenile forearm length for bats in the Western Montane region, but we still observed a significant decline over time. Wind speed may affect bat foraging and insect activity (Verboom and Spoelstra 1999; Møller 2013) but consistent daily wind speed data from nearby weather stations were not available. We were also unable to directly quantify prey abundance or availability in our study regions because no such data are available. Decoupling the energetic impacts of food supply from the impacts of weather on foraging activity is especially important in the context of significant and widespread insect decline (Wagner et al. 2021; Hallmann et al. 2017). More recent, large‐scale analyses of aerial arthropod abundance over time reveal considerable heterogeneity among regions and taxa, characterized by stronger declines among nocturnal species, and in high‐latitude regions experiencing pronounced winter warming (Mungee et al. 2025; Tielens et al. 2025). Consequently, nocturnal aerial insectivores in northern regions may be disproportionately affected by such reductions in food supply. Insect activity is correlated with changing temperature and rainfall regimes (Welti et al. 2022); thus, we are not able to disentangle the effect of food supply from the effect of local weather conditions relative to their impacts on bat body condition, but food supply must be a proximate cause of declining body size in insectivorous bats. In Bechstein's bat ( *Myotis bechsteinii* ), metabolic savings from warmer temperatures drives variation in body size more than food supply, and in other species, food supply has been found to influence daily torpor expression (Matheson et al. 2010; Wojciechowski et al. 2007; Mundinger et al. 2023). Other geographic differences in little brown bat behaviour such as diet, foraging habitat, and roost site selection (Clare et al. 2014; Sheridan et al. 2022; Jung et al. 2014) may also create contradictory patterns of energetic stress.

Although we were unable to incorporate arthropod abundance in our models, the available evidence suggests that ongoing landscape modification in our study regions could be causing arthropod declines. Land cover change can affect arthropod abundance and diversity (Attwood et al. 2008; Novotný et al. 2015; Seibold et al. 2019), especially where pesticide application for agriculture, forestry and other purposes is intense (Sohlström et al. 2022; Raven and Wagner 2021). The potential impacts of wildfires on bat body condition are also largely unquantified, but should not be discounted. Intensive forestry practices, invasive pest insects, and climate change have interactive effects on the frequency and severity of wildfires (Halofsky et al. 2020; Ibáñez et al. 2025). Increased wildfire frequency and severity can reduce microhabitat availability and insect abundance, and disrupt torpor (Ancillotto et al. 2021; Dole et al. 2023; Doty et al. 2018; Jung 2020). These short‐term, negative impacts may lead to increased chronic stress (Wasserman and Mueller 2023). Incorporating the effects of wildfires, land cover change or pesticide application is beyond the scope of this study, particularly as robust data on arthropod abundance are not available for our study areas. Nevertheless, these factors should be considered where possible in future work.

Despite the phenological and geographical variation in drivers of bat body mass, signatures of energetic stress in little brown bats are present continent‐wide, which likely has implications for population viability. In adult females, decreased body mass during pre‐ and mid‐hibernation periods has been associated with later emergence (Czenze and Willis 2015; Norquay and Willis 2014) which in turn delays parturition, and reduces survival of late‐born juveniles who may not be able to acquire sufficient resources for winter survival (Frick et al. 2010). We observed that juvenile bats were smaller, on average (i.e., had shorter forearms) in years when adult females emerged from hibernation with lower average body mass. This finding is consistent with previous results from the Yukon, where shorter forearms were associated with decreased odds of survival (Davy et al. 2022), possibly because higher surface to volume ratios reduce heat and water retention (Conenna et al. 2021; Rubalcaba et al. 2022) and smaller bodied animals are less able to store fat than larger bodied individuals. Females captured during the lactation period were apparently able to buffer their young from nutritional stress to some extent. However, this protection ended once young were weaned and was still not sufficient to prevent temporal patterns of decline in juvenile mass or in forearm length at maturity (Davy et al. 2022).

Poor body condition also has implications for immune response and resistance to disease. In some sites, survivors of bat white nose disease have higher fat stores, on average, compared to bats measured pre‐invasion (Cheng et al. 2019; Haase et al. 2021; Lacki et al. 2015). Our data, which represent a pre‐WND context, imply ongoing, widespread energetic stress that could promote high mortality rates for populations in the path of approaching WND invasion.

In conclusion, our parallel analyses of data from distinct geographic regions revealed consistent temporal trends in forearm length of bats across a wide geographic area and identified phenophases and weather conditions that may be energetically limiting for bats in the face of ongoing climate change. Our study improves the mechanistic understanding of climate‐driven energetic stress in little brown bats but highlights the need for more species‐ and location‐specific studies to further explore the complex and often conflicting physiological effects of climate change (Alston et al. 2023; Findlay‐Robinson et al. 2023; Kerth and Wolf 2025; Mundinger et al. 2023). Increased monitoring of body condition throughout the active season will further reveal the seasonally varying drivers of energetic stress in bats, particularly in northern and western North America, where swarming sites and hibernacula are difficult to find or access (Weller et al. 2018). Expanding data collection to include indices of food availability including factors that affect insect abundance such as quantifying pest control and habitat loss, could help make direct links between bat body condition and ecosystem management. The importance of tracking population size estimates, through roost monitoring or relative abundance models (e.g., North American Bat Monitoring Program; Loeb et al. 2015), will increase as we strive to understand the long‐term consequences of climate change and other stressors on bats.

## Author Contributions

**Christina M. Davy:** conceptualization, supervision, writing – review and editing. **Cori L. Lausen:** investigation, writing – review and editing. **Craig K. R. Willis:** investigation, writing – review and editing. **Valerie von Zuben:** conceptualization, data curation, formal analysis, writing – original draft, writing – review and editing. **Thomas S. Jung:** investigation, writing – review and editing. **Hugh G. Broders:** investigation, writing – review and editing. **Kaleigh J. O. Norquay:** investigation.

## Funding

University of Winnipeg Bat Lab funded by Natural Sciences and Engineering Research Council of Canada, Canada Foundation for Innovation, Government of Ontario, the Ontario Species at Risk Stewardship Program, U.S. Fish and Wildlife Service, Province of Manitoba; Yukon Bat Research Program funded by Government of Canada and Government of Yukon; Wildlife Conservation Society Canada Bat Program funded in part by British Columbia Government, Fish and Wildlife Compensation Program, Habitat Conservation Trust Foundation, and Forest Enhancement Society of BC; Broders Lab funded by the University of Waterloo, Saint Mary's University, Natural Sciences and Engineering Research Council of Canada, Parks Canada, The Canadian Wildlife Federation, the Department of Environment and Wildlife of the Province of Newfoundland and Labrador, Nova Scotia Species at Risk Conservation Fund, and the Nova Scotia Habitat Conservation Fund (Contributions from Hunters and Trappers).

## Conflicts of Interest

The authors declare no conflicts of interest.

## Supporting information


**Table S1:** Parameter estimates (*β*), 95% confidence intervals (CI), and marginal semi‐partial *R* ^2^ from linear mixed‐effects models examining focal and non‐focal predictors of average log body mass of adult female little brown bats ( *Myotis lucifugus* ) measured during each phenophase of the active season in five geographic regions. Bold text indicates variables with confidence intervals not overlapping zero.
**Table S2:** Parameter estimates (β), 95% confidence intervals (CI), and marginal semi‐partial R2 from mixed‐effects models examining focal and non‐focal predictors of average log body mass of adult male little brown bats (Myotis lucifugus) measured during each phenophase of the active season in five geographic regions. Bold text indicates variables with confidence intervals not overlapping zero.
**Table S3:** Parameter estimates, 95% confidence intervals, and marginal semi‐partial R2 from mixed‐effects models examining focal and non‐focal predictors of average log body mass of juvenile little brown bats (Myotis lucifugus) measured during each phenophase of the active season in five geographic regions. Bold text indicates variables with confidence intervals not overlapping zero.
**Table S4:** Parameter estimates (β) and 95% confidence intervals (CI) from linear regression models examining regional trends over each study period in (a) mean minimum temperature change per year and (b) change in number of rain days per year for each of the five phenophases of the adult female little brown bat (Myotis lucifugus) active season in each geographic region. Each observation is a mean minimum temperature or total number of rain days from each station, each phenophase, and each year. Bold text indicates variables with confidence intervals not overlapping zero.
**Figure S1:** Generalized additive model fits (and shaded 95% confidence intervals) examining the change in body mass of little brown bat (Myotis lucifugus) over the active season and identifying important inflection points (vertical lines) associated with biologically meaningful phenophases such as gestation, lactation, and swarming. Body mass is centred on the mean.
**Figure S2:** Linear mixed‐effects model fits (mean and shaded 95% confidence interval) showing the interactive effects of the proportion of rain days on log body mass (g) of little brown bats (Myotis lucifugus) at three levels (−1 SD, Mean, +1 SD) of proportion of warmer than average days among phenophases.
**Figure S3:** Parameter estimates (open dots) and 95% confidence intervals (bars) from linear mixed‐effect models predicting the effects of year on mean minimum temperature (top panel) and total rain days (bottom panel) for each active season phenophase of an adult female little brown bat (Myotis lucifugus) for each geographic region and respective study period. Confidence intervals are smaller than the point size when not visible. Mean minimum temperature and the total number of rain days increased over the study period when the parameter estimates are above zero, and decreased when estimates are below zero.

## Data Availability

Data and code (von Zuben 2026) are available in the Open Science Framework repository at https://osf.io/t2p5r/overview?view_only=53726c9dfd974249aa913b899d536d6c.
