# Supplementary material for: Body Size Decline in an Endangered Bat Is Associated With Climate Change at a Continental Scale but Varies by Phenophase and Region
Source: Glob Chang Biol. 2026 Jul 14;32(7):e70983. doi: 10.1111/gcb.70983 (PMC13366146; doi:10.1111/gcb.70983)
Supplement: Supplementary file 1 — Table S1: Parameter estimates (β), 95% confidence intervals (CI), and marginal semi‐partial R 2 from linear mixed‐effects models examining focal and non‐focal predictors of average log body mass of adult female little brown bats ( Myotis lucifugus ) measured during each phenophase of the active season in five geographic regions. Bold text indicates variables with confidence intervals not overlapping zero. Table S2: Parameter estimates (β), 95% confidence intervals (CI), and marginal semi‐partial R2 from mixed‐effects models examining focal and non‐focal predictors of average log body mass of adult male little brown bats (Myotis lucifugus) measured during each phenophase of the active season in five geographic regions. Bold text indicates variables with confidence intervals not overlapping zero. Table S3: Parameter estimates, 95% confidence intervals, and marginal semi‐partial R2 from mixed‐effects models examining focal and non‐focal predictors of average log body mass of juvenile little brown bats (Myotis lucifugus) measured during each phenophase of the active season in five geographic regions. Bold text indicates variables with confidence intervals not overlapping zero. Table S4: Parameter estimates (β) and 95% confidence intervals (CI) from linear regression models examining regional trends over each study period in (a) mean minimum temperature change per year and (b) change in number of rain days per year for each of the five phenophases of the adult female little brown bat (Myotis lucifugus) active season in each geographic region. Each observation is a mean minimum temperature or total number of rain days from each station, each phenophase, and each year. Bold text indicates variables with confidence intervals not overlapping zero. Figure S1: Generalized additive model fits (and shaded 95% confidence intervals) examining the change in body mass of little brown bat (Myotis lucifugus) over the active season and identifying important inflection points (vertical [file GCB-32-e70983-s001.docx]

**Supporting Information**

**Body size decline in an endangered bat is associated with climate change at a continental scale but varies by phenophase and region. Global Change Biology**

Valerie von Zuben^1^, Hugh G. Broders^2^, Thomas S. Jung^3,4^, Cori L. Lausen^5^, Kaleigh J. O. Norquay^6^, Craig K.R. Willis^6^, Christina M. Davy^7^

1 Wildlife Research and Monitoring Section, Ontario Ministry of Natural Resources, Peterborough, CAN; 2 Department of Biology, University of Waterloo, Waterloo, CAN; 3 Department of Environment, Government of Yukon, Whitehorse, CAN; 4 Department of Renewable Resources, University of Alberta, Edmonton, CAN; 5 Western Canada Bat Program, Wildlife Conservation Society Canada, Kaslo, CAN; 6 Department of Biology, University of Winnipeg, Winnipeg, CAN; 7 Department of Biology, Carleton University, Ottawa, CAN

**Table S1** Parameter estimates (β), 95% confidence intervals (CI), and marginal semi-partial R^2^ from linear mixed-effects models examining focal and non-focal predictors of average log body mass of adult female little brown bats (*Myotis lucifugus*) measured during each phenophase of the active season in five geographic regions. Bold text indicates variables with confidence intervals not overlapping zero.

|  |  | **Emergence** | | | | |  | **Gestation** | | | |  | **Lactation** | | | | |  | **Early swarming** | | |  | **Late swarming** | | |
| --- | --- | --- | --- | --- | --- | --- | --- | --- | --- | --- | --- | --- | --- | --- | --- | --- | --- | --- | --- | --- | --- | --- | --- | --- | --- |
|  |  |  | | | | |  |  | | | |  |  | | | | |  |  | | |  |  | | |
| **Yukon** |  | n obs. = 90 sites = 2 | | | | |  | n obs. = 2332 sites = 10 | | | |  | n obs. = 2274 sites = 11 | | | | |  | n obs. = 1428 sites = 10 | | |  | n = 0 | | |
|  |  | β | | CI | | R^2^ |  | β | CI | | R^2^ |  | β | | CI | | R^2^ |  | β | CI | R^2^ |  | β | CI | R^2^ |
|  | PropRain* | 0.002 | | -0.003, 0.007 | | 0.008 |  | -0.001 | **-0.002, -0.000** | | 0.000 |  | **-0.001** | | **-0.002, -0.000** | | 0.000 |  | - | - | 0.000 |  | - | - | - |
|  | PropWarm* | **-0.007** | | **-0.012, -0.003** | | 0.104 |  | -0.000 | -0.001, 0.000 | | 0.000 |  | -0.000 | | -0.001, 0.000 | | 0.000 |  | - | - | 0.039 |  | - | - | - |
|  | PropRain x PropWarm* | NS | NS | | - | |  | NS | NS | - | |  | NS | NS | | - | |  | **-0.000** | **-0.000, -0.000** | 0.004 |  | - | - | - |
|  | -1 SD | - | - | | - | |  | - | - | - | |  | - | - | | - | |  | **0.003** | **0.001, 0.004** | - |  | - | - | - |
|  | Mean | - | | - | | - |  | - | - | | - |  | - | | - | | - |  | **0.001** | **0.000, 0.002** | - |  | - | - | - |
|  | +1 SD | - | | - | | - |  | - | - | | - |  | - | | - | | - |  | -0.000 | -0.002., 0.001 | - |  | - | - | - |
|  | Year* | -0.002 | | -0.005, 0.002 | | 0.009 |  | **-0.011** | **-0.013, -0.009** | | 0.008 |  | **0.003** | | **0.001, 0.005** | | 0.003 |  | 0.000 | -0.002, 0.002 | 0.000 |  | - | - | - |
|  | Ordinal day | -** | | -** | | -** |  | **0.004** | **0.004, 0.005** | | 0.062 |  | **0.003** | | **0.002, 0.004** | | 0.004 |  | **0.001** | **0.001, 0.002** | 0.015 |  | - | - | - |
|  | 1^st^ order | NS | | NS | | - |  | NS | NS | | - |  | NS | | NS | | - |  | NS | NS | - |  | - | - | - |
|  | 2^nd^ order | NS | | NS | | - |  | NS | NS | | - |  | NS | | NS | | - |  | NS | NS | - |  | - | - | - |
|  | Forearm length | **0.016** | | **0.006, 0.027** | | 0.098 |  | **0.018** | **0.015, 0.022** | | 0.023 |  | **0.018** | | **0.014, 0.022** | | 0.016 |  | **0.020** | **0.016, 0.024** | 0.045 |  | - | - | - |
|  | Pregnant (yes) | - | | - | | - |  | **0.160** | **0.148, 0.172** | | 0.172 |  | **0.203** | | **0.194, 0.213** | | 0.391 |  | **0.120** | **0.105, 0.0136** | 0.131 |  | - | - | - |
|  |  |  | |  | |  |  |  |  | |  |  |  | |  | |  |  |  |  |  |  |  |  |  |
| **Western**  **Montane** |  |  | | n = 0 | |  |  | n obs. = 348 sites = 27 | | | |  | n obs. = 399 sites = 19 | | | | |  | n obs. = 131 sites = 11 | | |  |  | n = 0 |  |
|  |  | β | | CI | | R^2^ |  | β | CI | | R^2^ |  | β | | CI | | R^2^ |  | β | CI | R^2^ |  | β | CI | R^2^ |
|  | PropRain* | - | | - | | - |  | -0.003 | -0.008, 0.003 | | 0.000 |  | - | | - | | 0.000 |  | -0.002 | -0.008, 0.004 | 0.000 |  | - | - | - |
|  | PropWarm* | - | | - | | - |  | -0.000 | -0.003, 0.002 | | 0.000 |  | - | | - | | 0.000 |  | -0.001 | -0.006, 0.005 | 0.000 |  | - | - | - |
|  | PropRain x PropWarm* | - | | - | | - |  | NS | NS | | - |  | **0.000** | | **0.000, 0.001** | | 0.074 |  | NS | NS | - |  | - | - | - |
|  | -1 SD | - | | - | | - |  | - | - | | - |  | **-0.009** | | **-0.016, -0.003** | | - |  | - | - | - |  | - | - | - |
|  | Mean | - | | - | | - |  | - | - | | - |  | **-0.006** | | **-0.012, -0.001** | | - |  | - | - | - |  | - | - | - |
|  | +1 SD | - | | - | | - |  | - | - | | - |  | -0.003 | | -0.009, 0.002 | | - |  | - | - | - |  | - | - | - |
|  | Year* | - | | - | | - |  | **-0.011** | **-0.019, -0.004** | | 0.067 |  | -0.020 | | -0.042, 0.003 | | 0.112 |  | **-0.036** | **-0.055, -0.018** | 0.354 |  | - | - | - |
|  | Ordinal day | - | | - | | - |  | 0.001 | -0.001, 0.003 | | 0.022 |  | -0.003 | | -0.010, 0.004 | | 0.000 |  | -0.001 | -0.005, 0.002 | 0.000 |  | - | - | - |
|  | 1^st^ order | - | | - | | - |  | NS | NS | | - |  | NS | | NS | | - |  | NS | NS | - |  | - | - | - |
|  | 2^nd^ order | - | | - | | - |  | NS | NS | | - |  | NS | | NS | | - |  | NS | NS | - |  | - | - | - |
|  | Forearm length | - | | - | | - |  | **0.027** | **0.016, 0.037** | | 0.021 |  | **0.027** | | **0.016, 0.039** | | 0.081 |  | **0.031** | **0.016, 0.046** | 0.000 |  | - | - | - |
|  | Pregnant (yes) | - | | - | | - |  | **0.118** | **0.093, 0.143** | | 0.166 |  | **0.157** | | **0.120, 0.194** | | 0.059 |  |  | - |  |  | - | - | - |
|  |  |  | |  | |  |  |  |  | |  |  |  | |  | |  |  |  |  |  |  |  |  |  |
| **Central**  **Canada** |  | n obs. = 625 sites = 13 | | | | |  | n obs. = 406 sites = 19 | | | |  | n obs. = 486 sites = 21 | | | | |  | n obs. = 876 sites = 18 | | |  | n obs. = 866 sites = 9 | | |
|  |  | β | | CI | | R^2^ |  | β | CI | | R^2^ |  | β | | CI | | R^2^ |  | β | CI | R^2^ |  | β | CI | R^2^ |
|  | PropRain* | **-0.002** | | **-0.004, -0.001** | | 0.066 |  | -0.001 | -0.005, 0.003 | | 0.000 |  | - | | - | | 0.000 |  | **-0.006** | **-0.008, -0.004** | 0.107 |  | - | - | 0.188 |
|  | PropWarm* | **-0.003** | | **-0.003, -0.002** | | 0.137 |  | **-0.003** | **-0.005, -0.001** | | 0.071 |  | - | | - | | 0.033 |  | 0.001 | -0.000, 0.002 | 0.000 |  | - | - | 0.000 |
|  | PropRain x PropWarm* | NS | | NS | | - |  | NS | NS | | - |  | **0.001** | | **0.001, 0.002** | | 0.000 |  | NS | NS | - |  | **0.001** | **0.000, 0.001** | 0.000 |
|  | -1 SD | - | | - | | - |  | - | - | | - |  | **-0.011** | | **-0.017, -0.006** | | - |  | - | - | - |  | **-0.019** | **-0.022, -0.016** | - |
|  | Mean | - | | - | | - |  | - | - | | - |  | -0.001 | | -0.005, 0.002 | | - |  | - | - | - |  | **-0.010** | **-0.014, -0.005** | - |
|  | +1 SD | - | | - | | - |  | - | - | | - |  | **0.009** | | **0.002, 0.016** | | - |  | - | - | - |  | -0.001 | -0.010, 0.008 | - |
|  | Year* | **-0.011** | | **-0.002, -0.000** | | 0.038 |  | -0.007 | -0.023, 0.010 | | 0.025 |  | 0.003 | | -0.005, 0.012 | | 0.000 |  | -0.004 | -0.009, 0.000 | 0.018 |  | **-0.010** | **-0.016, -0.003** | 0.043 |
|  | Ordinal day | **-0.004** | | **-0.006, -0.003** | | 0.099 |  | **0.004** | **0.001, 0.006** | | 0.019 |  | **0.003** | | **0.000, 0.005** | | 0.032 |  | -0.001 | -0.002, 0.000 | 0.022 |  | - | - | 0.121 |
|  | 1^st^ order | NS | | NS | | - |  | NS | NS | | - |  | NS | | NS | | - |  | NS | NS | - |  | **2.359** | **2.084, 2.635** | - |
|  | 2^nd^ order | NS | | NS | | - |  | NS | NS | | - |  | NS | | NS | | - |  | NS | NS | - |  | **-1.313** | **-1.553, -1.074** | - |
|  | Forearm length | **0.027** | | **0.019, 0.035** | | 0.000 |  | **0.021** | **0.013, 0.030** | | 0.037 |  | **0.024** | | **0.015, 0.033** | | 0.038 |  | **0.025** | **0.018, 0.031** | 0.063 |  | **0.022** | **0.014, 0.030** | 0.022 |
|  | Pregnant (yes) | - | | - | | - |  | **0.159** | **0.131, 0.186** | | 0.262 |  | **0.133** | | **0.052, 0.214** | | 0.017 |  | **0.147** | **0.081, 0.214** | 0.022 |  | - | - | - |
|  |  |  | |  | |  |  |  |  | |  |  |  | |  | |  |  |  |  |  |  |  |  |  |
| **Southern**  **Maritimes** |  |  | | n = 0 | |  |  | n obs. = 385 sites = 15 | | | |  | n obs. = 113 sites = 6 | | | | |  | n obs. = 291 sites = 27 | | |  | n obs. = 306 sites = 15 | | |
|  |  | β | | CI | | R^2^ |  | β | CI | | R^2^ |  | β | | CI | | R^2^ |  | β | CI | R^2^ |  | β | CI | R^2^ |
|  | PropRain* | - | | - | | - |  | -0.001 | -0.004, 0.002 | | 0.032 |  | 0.008 | | -0.005, 0.021 | | 0.075 |  | 0.003 | -0.001, 0.007 | 0.032 |  | -0.001 | -0.004, 0.002 | 0.005 |
|  | PropWarm* | - | | - | | - |  | -0.001 | -0.003, 0.002 | | 0.013 |  | -0.004 | | -0.021, 0.013 | | 0.056 |  | 0.001 | -0.001, 0.004 | 0.003 |  | **-0.003** | **-0.005, -0.000** | 0.000 |
|  | PropRain x PropWarm* | - | | - | | - |  | NS | NS | | - |  | NS | | NS | | - |  | NS | NS | - |  | NS | NS | - |
|  | -1 SD | - | | - | | - |  | - | - | | - |  | - | | - | | - |  | - | - | - |  | - | - | - |
|  | Mean | - | | - | | - |  | - | - | | - |  | - | | - | | - |  | - | - | - |  | - | - | - |
|  | +1 SD | - | | - | | - |  | - | - | | - |  | - | | - | | - |  | - | - | - |  | - | - | - |
|  | Year* | - | | - | | - |  | -0.001 | -0.011, 0.010 | | 0.000 |  | **0.041** | | **0.016, 0.066** | | 0.140 |  | -0.005 | -0.013, 0.004 | 0.030 |  | 0.001 | -0.009, 0.012 | 0.001 |
|  | Ordinal day | - | | - | | - |  | **0.007** | **0.005, 0.009** | | 0.000 |  | 0.002 | | -0.009, 0.014 | | 0.000 |  | -0.001 | -0.003, 0.001 | 0.009 |  | - | - | 0.24 |
|  | 1^st^ order | - | | - | | - |  | NS | NS | | - |  | NS | | NS | | - |  | NS | NS | - |  | **1.312** | **0.997, 1.627** | - |
|  | 2^nd^ order | - | | - | | - |  | NS | NS | | - |  | NS | | NS | | - |  | NS | NS | - |  | **-0.755** | **-1.019, -0.491** | - |
|  | Forearm length | - | | - | | - |  | **0.030** | **0.020, 0.041** | | 0.011 |  | **0.048** | | **0.026, 0.069** | | 0.027 |  | **0.021** | **0.010, 0.033** | 0.033 |  | **0.034** | **0.022, 0.047** | 0.051 |
|  | Pregnant (yes)† | - | | - | | - |  | - | - | | - |  | - | | - | | - |  | - | - | - |  | - | - | - |
|  |  |  | |  | |  |  |  |  | |  |  |  | |  | |  |  |  |  |  |  |  |  |  |
| **Newfoundland** |  | n obs. = 404 sites = 2 | | | | |  | n obs. = 414 sites = 3 | | | |  | n obs. = 420 sites = 4 | | | | |  | n obs. = 351 sites = 4 | | |  |  | n = 0 |  |
|  |  | β | | CI | | R^2^ |  | β | CI | | R^2^ |  | β | | CI | | R^2^ |  | β | CI | R^2^ |  | β | CI | R^2^ |
|  | PropRain* | - | | - | | 0.026 |  | - | - | | 0.031 |  | - | | - | | 0.033 |  | - | - | 0.014 |  | - | - | - |
|  | PropWarm* | - | | - | | 0.031 |  | - | - | | 0.046 |  | - | | - | | 0.041 |  | - | - | 0.011 |  | - | - | - |
|  | PropRain x PropWarm* | **-0.001** | | **-0.001, -0.000** | | 0.028 |  | **0.001** | **0.000, 0.001** | | 0.030 |  | **0.000** | | **0.000, 0.001** | | 0.034 |  | **-0.000** | **-0.000, -0.000** | **0.015** |  | - | - | - |
|  | -1 SD | **0.008** | | **0.002, 0.013** | | - |  | **-0.003** | **-0.005, -0.002** | | - |  | -0.000 | | -0.001, 0.001 | | - |  | 0.001 | -0.000, 0.003 | - |  | - | - | - |
|  | Mean | 0.002 | | -0.000, 0.004 | | - |  | 0.001 | -0.000, 0.002 | | - |  | **0.002** | | **0.001, 0.004** | | - |  | -0.000 | -0.002, 0.001 | - |  | - | - | - |
|  | +1 SD | **-0.004** | | **-0.006, -0.002** | | - |  | **0.005** | **0.002, 0.009** | | - |  | **0.005** | | **0.003, 0.008** | | - |  | **-0.002** | **-0.005, -0.000** | - |  | - | - | - |
|  | Year* | -*** | | -*** | | - |  | -*** | -*** | | - |  | -*** | | -*** | | - |  | -*** | -*** | - |  | - | - | - |
|  | Ordinal day | 0.003 | | -0.002, 0.007 | | 0.003 |  | **0.007** | **0.006, 0.008** | | 0.355 |  | **-0.007** | | **-0.009, -0.004** | | 0.073 |  | -0.001 | -0.004, 0.003 | 0.004 |  | - | - | - |
|  | 1^st^ order | NS | | NS | | - |  | NS | NS | | - |  | NS | | NS | | - |  | NS | NS | - |  | - | - | - |
|  | 2^nd^ order | NS | | NS | | - |  | NS | NS | | - |  | NS | | NS | | - |  | NS | NS | - |  | - | - | - |
|  | Forearm length | **0.018** | | **0.012, 0.024** | | 0.087 |  | **0.020** | **0.009, 0.031** | | 0.030 |  | **0.027** | | **0.017, 0.037** | | 0.063 |  | **0.017** | **0.009, 0.025** | 0.050 |  | - | - | - |
|  | Pregnant (yes)† | - | | - | | - |  | - | - | | - |  | - | | - | | - |  | - | - | - |  | - | - | - |
|  |  |  | |  | |  |  |  |  | |  |  |  | |  | |  |  |  |  |  |  |  |  |  |

*Focal variables ** Removed variable; collinearity † Variable not assessed

Abbreviations: PropRain, proportion of rain days prior to capture; PropWarm, proportion of warmer-than-average days prior to capture; NS, not significant (p ≥ 0.05); dash, not applicable

**Table S2** Parameter estimates (β), 95% confidence intervals (CI), and marginal semi-partial R^2^ from mixed-effects models examining focal and non-focal predictors of average log body mass of adult male little brown bats (*Myotis lucifugus*) measured during each phenophase of the active season in five geographic regions. Bold text indicates variables with confidence intervals not overlapping zero.

|  |  | **Emergence** | | |  | **Summer activity** | | |  | **Early swarming** | | |  | **Late swarming** | | |
| --- | --- | --- | --- | --- | --- | --- | --- | --- | --- | --- | --- | --- | --- | --- | --- | --- |
|  |  |  | | |  |  | | |  |  | | |  |  | | |
| **Yukon** |  | n = 0 | | |  | n = 0 | | |  | n = 0 | | |  | n = 0 | | |
|  |  |  | | |  |  | | |  |  | | |  |  | | |
|  |  |  |  |  |  |  |  |  |  |  |  |  |  |  |  |  |
| **Western**  **Montane** |  | n = 0 | | |  | n obs. = 262 sites = 31 | | |  | n = 0 | | |  | n obs. = 91 sites = 8 | | |
|  |  | β | CI | R^2^ |  | β | CI | R^2^ |  | β | CI | R^2^ |  | β | CI | R^2^ |
|  | PropRain* | - | - | - |  | - | - | 0.050 |  | - | - | - |  | 0.012 | -0.019, 0.042 | 0.008 |
|  | PropWarm* | - | - | - |  | - | - | 0.011 |  | - | - | - |  | -0.006 | -0.027, 0.015 | 0.065 |
|  | PropRain x PropWarm* | - | - | - |  | **-0.000** | **-0.000, -0.000** | 0.049 |  | - | - | - |  | NS | NS | - |
|  | -1 SD | - | - | - |  | **0.006** | **0.002, 0.009** | - |  | - | - | - |  | - | - | - |
|  | Mean | - | - | - |  | **0.003** | **0.000, 0.006** | - |  | - | - | - |  | - | - | - |
|  | +1 SD | - | - | - |  | 0.000 | -0.003, 0.004 | - |  | - | - | - |  | - | - | - |
|  | Year* | - | - | - |  | 0.000 | -0.007, 0.008 | 0.001 |  | - | - | - |  | 0.016 | -0.029, 0.060 | 0.100 |
|  | Ordinal day | - | - | - |  | **0.003** | **0.002, 0.004** | 0.055 |  | - | - | - |  | -0.000 | -0.014, 0.013 | 0.000 |
|  | 1^st^ order | - | - | - |  | NS | NS | - |  | - | - | - |  | NS | NS | - |
|  | 2^nd^ order | - | - | - |  | NS | NS | - |  | - | - | - |  | NS | NS | - |
|  | Forearm length | - | - | - |  | **0.031** | **0.021, 0.042** | 0.095 |  | - | - | - |  | 0.020 | -0.001, 0.040 | 0.000 |
|  |  |  |  |  |  |  |  |  |  |  |  |  |  |  |  |  |
| **Central**  **Canada** |  | n obs. = 920 sites = 10 | | |  | n obs. = 769 sites = 9 | | |  | n obs. = 655 sites = 10 | | |  | n obs. = 1168 sites = 9 | | |
|  |  | β | CI | R^2^ |  | β | CI | R^2^ |  | β | CI | R^2^ |  | β | CI | R^2^ |
|  | PropRain* | **-0.003** | **-0.004, -0.002** | 0.043 |  | **-0.007** | **-0.008, -0.005** | 0.177 |  | **-0.003** | **-0.006, -0.001** | 0.030 |  | **-0.020** | **-0.023, -0.017** | 0.283 |
|  | PropWarm* | **-0.001** | **-0.002, -0.000** | 0.018 |  | -0.000 | -0.001,0.001 | 0.000 |  | **0.002** | **0.001, 0.004** | 0.005 |  | **0.004** | **0.003, 0.006** | 0.000 |
|  | PropRain x PropWarm* | NS | NS | - |  | NS | NS | - |  | NS | NS | - |  | NS | NS | - |
|  | -1 SD | - | - | - |  | - | - | - |  | - | - | - |  | - | - | - |
|  | Mean | - | - | - |  | - | - | - |  | - | - | - |  | - | - | - |
|  | +1 SD | - | - | - |  | - | - | - |  | - | - | - |  | - | - | - |
|  | Year* | **-0.030** | **-0.040, -0.020** | 0.049 |  | **-0.022** | **-0.029, -0.014** | **0.095** |  | 0.001 | -0.006, 0.007 | 0.000 |  | **-0.030** | **-0.039, -0.020** | 0.130 |
|  | Ordinal day | **-0.008** | **-0.009, -0.007** | 0.103 |  | - | - | 0.309 |  | - | - | 0.042 |  | - | - | 0.000 |
|  | 1^st^ order | NS | NS | - |  | **3.084** | **2.644, 3.525** | - |  | **-0.574** | **-0.856, -0.292** | - |  | **2.010** | **1.667, 2.353** | - |
|  | 2^nd^ order | NS | NS | - |  | **-2.226** | **-2.496, -1.956** | - |  | **-0.360** | **-0.610, -0.111** | - |  | **-0.749** | **-1.033, -0.466** | - |
|  | Forearm length | **0.023** | **0.017, 0.029** | 0.016 |  | **0.025** | **0.018, 0.033** | 0.050 |  | **0.027** | **0.018, 0.035** | 0.058 |  | **0.027** | **0.020, 0.034** | 0.027 |
|  |  |  |  |  |  |  |  |  |  |  |  |  |  |  |  |  |
| **Southern**  **Maritimes** |  | n obs. = 211 sites = 6 | | | | n obs. = 222 sites = 18 | | |  | n obs. = 124 sites = 16 | | |  | n obs. = 685 sites = 17 | | |
|  |  | β | CI | R^2^ |  | β | CI | R^2^ |  | β | CI | R^2^ |  | β | CI | R^2^ |
|  | PropRain* | 0.002 | -0.000, 0.004 | 0.013 |  | 0.001 | -0.002, 0.003 | 0.000 |  | -0.000 | -0.003, 0.003 | 0.000 |  | -0.001 | -0.003, 0.002 | 0.001 |
|  | PropWarm* | 0.000 | -0.001, 0.001 | 0.003 |  | 0.001 | -0.000, 0.003 | 0.013 |  | 0.003 | -0.000, 0.005 | 0.000 |  | 0.001 | -0.001, 0.002 | 0.002 |
|  | PropRain x PropWarm* | NS | NS | - |  | NS | NS | - |  | NS | NS | - |  | NS | NS | - |
|  | -1 SD | - | - | - |  | - | - | - |  | - | - | - |  | - | - | - |
|  | Mean | - | - | - |  | - | - | - |  | - | - | - |  | - | - | - |
|  | +1 SD | - | - | - |  | - | - | - |  | - | - | - |  | - | - | - |
|  | Year* | -*** | -*** | - |  | -0.001 | -0.008, 0.005 | 0.008 |  | -0.004 | -0.017, 0.010 | 0.037 |  | -0.006 | -0.013, 0.002 | 0.000 |
|  | Ordinal day | 0.001 | -0.002, 0.004 | 0.006 |  | **0.004** | **0.003, 0.005** | 0.209 |  | 0.001 | -0.002, 0.003 | 0.000 |  | - | - | 0.165 |
|  | 1^st^ order | NS | NS | - |  | NS | NS | - |  | NS | NS | - |  | **1.517** | **1.190, 1.844** | - |
|  | 2^nd^ order | NS | NS | - |  | NS | NS | - |  | NS | NS | - |  | **-1.115** | **-1.432, -0.797** | - |
|  | Forearm length | **0.019** | **0.009, 0.030** | 0.045 |  | **0.015** | **0.007, 0.023** | 0.050 |  | **0.023** | **0.008, 0.039** | 0.022 |  | **0.023** | **0.012, 0.034** | 0.019 |
|  |  |  |  |  |  |  |  |  |  |  |  |  |  |  |  |  |
| **Newfoundland** |  |  | n = 0 |  |  |  | n = 0 |  |  |  | n = 0 |  |  |  | n = 0 |  |
|  |  |  |  |  |  |  |  |  |  |  |  |  |  |  |  |  |
|  |  |  |  |  |  |  |  |  |  |  |  |  |  |  |  |  |

*Focal variables *** Removed variable; insufficient range of years

Abbreviations: PropRain, proportion of rain days prior to capture; PropWarm, proportion of warmer-than-average days prior to capture; NS, not significant (p ≥ 0.05); dash, not applicable

**Table S3** Parameter estimates, 95% confidence intervals, and marginal semi-partial R^2^ from mixed-effects models examining focal and non-focal predictors of average log body mass of juvenile little brown bats (*Myotis lucifugus*) measured during each phenophase of the active season in five geographic regions. Bold text indicates variables with confidence intervals not overlapping zero.

|  |  | **Volancy** | | |  | **Early swarming** | | |  | **Late swarming** | | |
| --- | --- | --- | --- | --- | --- | --- | --- | --- | --- | --- | --- | --- |
|  |  |  | | |  |  | | |  |  | | |
| Yukon |  | n obs. = 143 sites = 5 | | |  | n obs. = 622 sites = 10 | | |  | n = 0 | | |
|  |  | β | CI | R^2^ |  | β | CI | R^2^ |  | β | CI | R^2^ |
|  | PropRain* | **-0.002** | **-0.003, -0.001** | 0.020 |  | **-0.002** | **-0.003, -0.000** | 0.008 |  | - | - | - |
|  | PropWarm* | -0.000 | -0.002, 0.002 | 0.000 |  | 0.001 | -0.000, 0.001 | 0.000 |  | - | - | - |
|  | PropRain x PropWarm* | NS | NS | - |  | NS | NS | - |  | - | - | - |
|  | -1 SD | - | - | - |  | - | - | - |  | - | - | - |
|  | Mean | - | - | - |  | - | - | - |  | - | - | - |
|  | +1 SD | - | - | - |  | - | - | - |  | - | - | - |
|  | Year* | **0.010** | **0.003, 0.016** | 0.050 |  | 0.001 | -0.002, 0.005 | 0.001 |  |  | - |  |
|  | Ordinal day | -** | -** | - |  | **0.003** | **0.001, 0.005** | 0.013 |  | - | - | - |
|  | 1^st^ order | - | - | - |  | NS | NS | - |  | - | - | - |
|  | 2^nd^ order | - | - | - |  | NS | NS | - |  | - | - | - |
|  | Forearm length | **0.052** | **0.039, 0.066** | 0.279 |  | **0.040** | **0.034, 0.046** | 0.166 |  | - | - | - |
|  | Sex (male) | -0.003 | -0.034, 0.028 | 0.000 |  | -0.023 | -0.037, -0.009 | 0.010 |  | - | - | - |
|  |  |  |  |  |  |  |  |  |  |  |  |  |
| Western  Montane |  |  | n = 0 |  |  | n obs. = 82 sites = 11 | | |  | n obs. = 60 sites = 11 | | |
|  |  | β | CI | R^2^ |  | β | CI | R^2^ |  | β | CI | R^2^ |
|  | PropRain* | - | - | - |  | -0.002 | -0.010, 0.007 | 0.000 |  | **0.013** | **0.007, 0.019** | 0.159 |
|  | PropWarm* | - | - | - |  | 0.000 | -0.003, 0.003 | 0.000 |  | 0.001 | -0.003, 0.004 | 0.000 |
|  | PropRain x PropWarm* | - | - | - |  | NS | NS | - |  | NS | NS | - |
|  | -1 SD | - | - | - |  | - | - | - |  | - | - | - |
|  | Mean | - | - | - |  | - | - | - |  | - | - | - |
|  | +1 SD | - | - | - |  | - | - | - |  | - | - | - |
|  | Year* | - | - | - |  | **-0.025** | **-0.041, -0.009** | 0.247 |  | **-0.026** | **-0.045, -0.008** | 0.000 |
|  | Ordinal day | - | - | - |  | **-0.006** | **-0.011, -0.000** | 0.053 |  | 0.008 | -0.000,0.017 | 0.097 |
|  | 1^st^ order | - | - | - |  | NS | NS | - |  | NS | NS | - |
|  | 2^nd^ order | - | - | - |  | NS | NS | - |  | NS | NS | - |
|  | Forearm length | - | - | - |  | **0.040** | **0.022, 0.058** | 0.187 |  | **0.037** | **0.019, 0.055** | 0.086 |
|  | Sex (male) | - | - | - |  | -0.020 | -0.071, 0.031 | 0.019 |  | -0.001 | -0.041, 0.040 | 0.000 |
|  |  |  |  |  |  |  |  |  |  |  |  |  |
| Central  Canada |  | n obs. = 297 sites = 24 | | |  | n obs. = 1377 sites = 15 | | |  | n obs. = 183 sites = 10 | | |
|  |  | β | CI | R^2^ |  | β | CI | R^2^ |  | β | CI | R^2^ |
|  | PropRain* | -0.002 | -0.004, 0.001 | 0.010 |  | 0.000 | -0.001, 0.002 | 0.000 |  | - | - | 0.000 |
|  | PropWarm* | -0.000 | -0.002, 0.001 | 0.000 |  | 0.000 | -0.000, 0.001 | 0.000 |  | - | - | 0.000 |
|  | PropRain x PropWarm* | NS | NS | - |  | NS | NS | - |  | **0.002** | **0.001, 0.002** | 0.236 |
|  | -1 SD | - | - | - |  | - | - | - |  | **-0.009** | **-0.014, -0.004** | - |
|  | Mean | - | - | - |  | - | - | - |  | 0.008 | -0.002, 0.017 | - |
|  | +1 SD | - | - | - |  | - | - | - |  | **0.024** | **0.006, 0.043** | - |
|  | Year* | 0.003 | -0.007, 0.012 | 0.000 |  | **0.009** | **0.005, 0.013** | 0.003 |  | -0.002 | -0.015, 0.010 | 0.000 |
|  | Ordinal day | -0.002 | -0.004, 0.000 | 0.006 |  | **0.003** | **0.003, 0.004** | 0.040 |  | - | - | 0.032 |
|  | 1^st^ order | NS | NS | - |  | NS | NS | - |  | **1.163** | **0.808, 1.519** | - |
|  | 2^nd^ order | NS | NS | - |  | NS | NS | - |  | **-0.816** | **-1.065, -0.567** | - |
|  | Forearm length | **0.034** | **0.024, 0.043** | 0.093 |  | **0.021** | **0.016, 0.026** | 0.038 |  | **0.025** | **0.012, 0.039** | 0.022 |
|  | Sex (male) | **-0.034** | **-0.056, -0.012** | 0.018 |  | **-0.021** | **-0.032, -0.011** | 0.010 |  | -0.012 | -0.038, 0.014 | 0.000 |
|  |  |  |  |  |  |  |  |  |  |  |  |  |
| Southern  Maritimes |  | n obs. = 175 | | sites = 19 |  | n obs. = 277 sites = 18 | | |  | n = 63†† | | |
|  |  | β | CI | R^2^ |  | β | CI | R^2^ |  | β | CI | R^2^ |
|  | PropRain* | -0.003 | -0.007, 0.001 | 0.028 |  | -0.000 | -0.002, 0.002 | 0.001 |  | - | - | - |
|  | PropWarm* | -0.002 | -0.005, 0.002 | 0.003 |  | **-0.001** | **-0.003, -0.000** | 0.030 |  | - | - | - |
|  | PropRain x PropWarm* | NS | NS | - |  | NS | NS | - |  | - | - | - |
|  | -1 SD | - | - | - |  | - | - | - |  | - | - | - |
|  | Mean | - | - | - |  | - | - | - |  | - | - | - |
|  | +1 SD | - | - | - |  | - | - | - |  | - | - | - |
|  | Year* | **-0.012** | **-0.023, -0.001** | 0.184 |  | 0.000 | -0.005, 0.006 | 0.001 |  | - | - | - |
|  | Ordinal day | -0.001 | -0.007,0.006 | 0.003 |  | 0.000 | -0.002, 0.002 | 0.000 |  | - | - | - |
|  | 1^st^ order | NS | NS | - |  | NS | NS | - |  | - | - | - |
|  | 2^nd^ order | NS | NS | - |  | NS | NS | - |  | - | - | - |
|  | Forearm length | **0.035** | **0.025, 0.046** | 0.126 |  | **0.023** | **0.014, 0.033** | 0.072 |  | - | - | - |
|  | Sex (male) | -0.001 | -0.027, 0.025 | 0.000 |  | -0.007 | -0.030, 0.015 | 0.002 |  | - | - | - |
|  |  |  |  |  |  |  |  |  |  |  |  |  |
| **Newfoundland** |  | n obs. = 221 sites = 4 | | | n obs. = 195 sites = 1 | | | |  |  | n = 0 |  |
|  |  | β | CI | R^2^ |  | β | CI | R^2^ |  | β | CI | R^2^ |
|  | PropRain* | 0.000 | -0.001 ,0.001 | 0.002 |  | **-0.004** | **-0.007, -0.001** | 0.046 |  | - | - | - |
|  | PropWarm* | **-0.001** | **-0.001, 0.000** | 0.053 |  | -0.000 | -0.001, 0.000 | 0.002 |  | - | - | - |
|  | PropRain x PropWarm* | NS | NS | - |  | NS | NS | - |  | - | - | - |
|  | -1 SD | - | - | - |  | - | - | - |  | - | - | - |
|  | Mean | - | - | - |  | - | - | - |  | - | - | - |
|  | +1 SD | - | - | - |  | - | - | - |  | - | - | - |
|  | Year* | -*** | -*** | -*** |  | -*** | -*** | - |  | - | - | - |
|  | Ordinal day | 0.002 | -0.000, 0.005 | 0.012 |  | **-0.008** | **-0.015, -0.002** | 0.031 |  | - | - | - |
|  | 1^st^ order | NS | NS | - |  | NS | NS | - |  | - | - | - |
|  | 2^nd^ order | NS | NS | - |  | NS | NS | - |  | - | - | - |
|  | Forearm length | **0.027** | **0.018, 0.035** | 0.152 |  | **0.030** | **0.020, 0.040** | 0.155 |  | - | - | - |
|  | Sex (male) | -0.008 | -0.026, 0.010 | 0.004 |  | -0.021 | -0.042, 0.000 | 0.020 |  | - | - | - |

*Focal variables ** Removed variable; collinearity *** Removed variable; insufficient range of years †† Model failed overfit test

Abbreviations: PropRain, proportion of rain days prior to capture; PropWarm, proportion of warmer-than-average days prior to capture; NS, not significant (p ≥ 0.05); dash, not applicable

**Table S4** Parameter estimates (β) and 95% confidence intervals (CI) from linear regression models examining regional trends over each study period in a) mean minimum temperature change per year and b) change in number of rain days per year for each of the five phenophases of the adult female little brown bat (*Myotis lucifugus*) active season in each geographic region. Each observation is a mean minimum temperature or total number of rain days from each station, each phenophase, and each year. Bold text indicates variables with confidence intervals not overlapping zero.

|  |  | **Yukon** | |  | **Western Montane** | |  | **Central Canada** | |  | **Southern Maritimes** | |  | **Newfoundland** | |
| --- | --- | --- | --- | --- | --- | --- | --- | --- | --- | --- | --- | --- | --- | --- | --- |
| Observations |  | n = 375 | |  | n = 2640 | |  | n = 1100 | |  | n = 1610 | |  | n = 200 | |
| Weather stations |  | n = 5 | |  | n = 33 | |  | n = 20 | |  | n = 23 | |  | n = 5 | |
| Study period (years) |  | n = 15 | |  | n = 16 | |  | n = 11 | |  | n = 14 | |  | n = 8 | |
| 1. Mean minimum temp. |  | β | CI |  | β | CI |  | β | CI |  | β | CI |  | β | CI |
| Emergence |  | 0.065 | -0.003, 0.134 |  | **0.068** | **0.047, 0.090** |  | 0.017 | -0.045, 0.079 |  | **0.114** | **0.081, 0.146** |  | -0.086 | -0.273, 0.102 |
| Gestation |  | -0.012 | -0.081, 0.056 |  | 0.018 | -0.003, 0.040 |  | **0.135** | **0.073, 0.197** |  | 0.024 | -0.008, 0.056 |  | -0.102 | -0.290, 0.085 |
| Lactation |  | 0.018 | -0.051, 0.087 |  | **-0.054** | **-0.076, -0.033** |  | **0.090** | **0.029, 0.152** |  | 0.020 | -0.012, 0.052 |  | -0.146 | -0.334, 0.041 |
| Early swarming |  | **0.079** | **0.011, 0.148** |  | **0.044** | **0.023, 0.066** |  | -0.050 | -0.112, 0.012 |  | 0.031 | -0.001, 0.063 |  | 0.136 | -0.052, 0.323 |
| Late swarming |  | **0.005** | -0.064, 0.073 |  | -0.002 | -0.023, 0.020 |  | **0.129** | **0.068, 0.191** |  | **0.042** | **0.009, 0.074** |  | -0.027 | -0.214, 0.160 |
| 1. Rain days |  | β | CI |  | β | CI |  | β | CI |  | β | CI |  | β | CI |
| Emergence |  | -0.078 | -0.257, 0.101 |  | **-0.100** | **-0.175, -0.026** |  | **-0.167** | **-0.305, -0.030** |  | **0.125** | **0.038, 0.213** |  | **-0.629** | **-1.148, -0.109** |
| Gestation |  | 0.050 | -0.129, 0.229 |  | **-0.280** | **-0.354, -0.205** |  | -0.086 | -0.224, 0.051 |  | **0.139** | **0.051, 0.226** |  | **-0.857** | **-1.376, -0.338** |
| Lactation |  | -0.001 | -0.181, 0.178 |  | 0.019 | -0.055, 0.094 |  | 0.071 | -0.066, 0.209 |  | -0.031 | -0.118, 0.057 |  | -0.183 | -0.703, 0.336 |
| Early swarming |  | 0.050 | -0.129, 0.229 |  | **-0.085** | **-0.160, -0.011** |  | **-0.164** | **-0.301, -0.026** |  | **0.224** | **0.137, 0.312** |  | -0.407 | -0.926, 0.112 |
| Late swarming |  | **-0.428** | **-0.607, -0.249** |  | -0.045 | -0.119, 0.030 |  | 0.115 | -0.023, 0.252 |  | **0.292** | **0.205, 0.379** |  | 0.045 | -0.474, 0.564 |

**
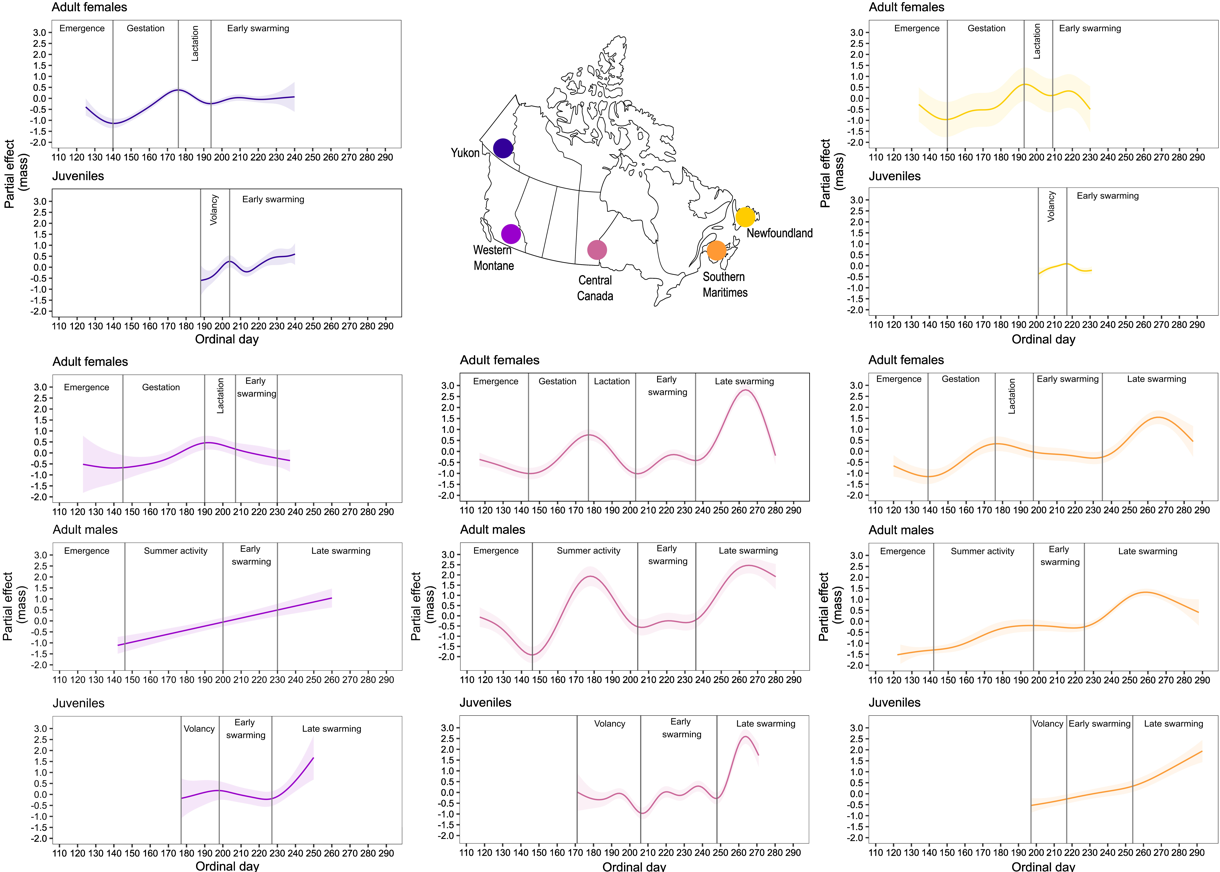
**

**Figure S1** Generalized additive model fits (and shaded 95% confidence intervals) examining the change in body mass of little brown bat (*Myotis lucifugus*) over the active season and identifying important inflection points (vertical lines) associated with biologically meaningful phenophases such as gestation, lactation, and swarming. Body mass is centered on the mean.


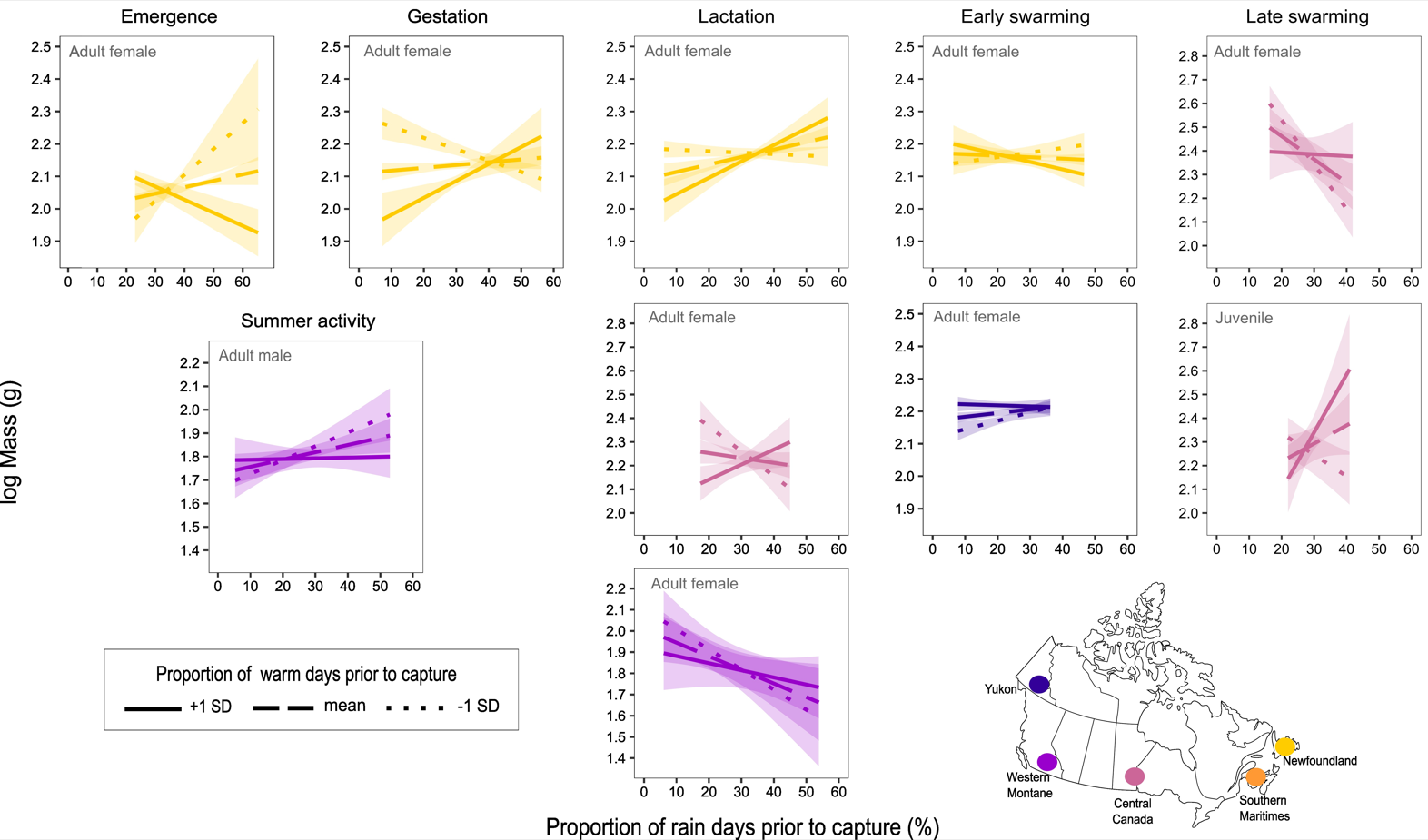


**Figure S2** Linear mixed-effects model fits (mean and shaded 95% confidence interval) showing the interactive effects of the proportion of rain days on log body mass (g) of little brown bats (*Myotis lucifugus*) at three levels (-1 SD, Mean, +1 SD) of proportion of warmer-than-average days among phenophases.


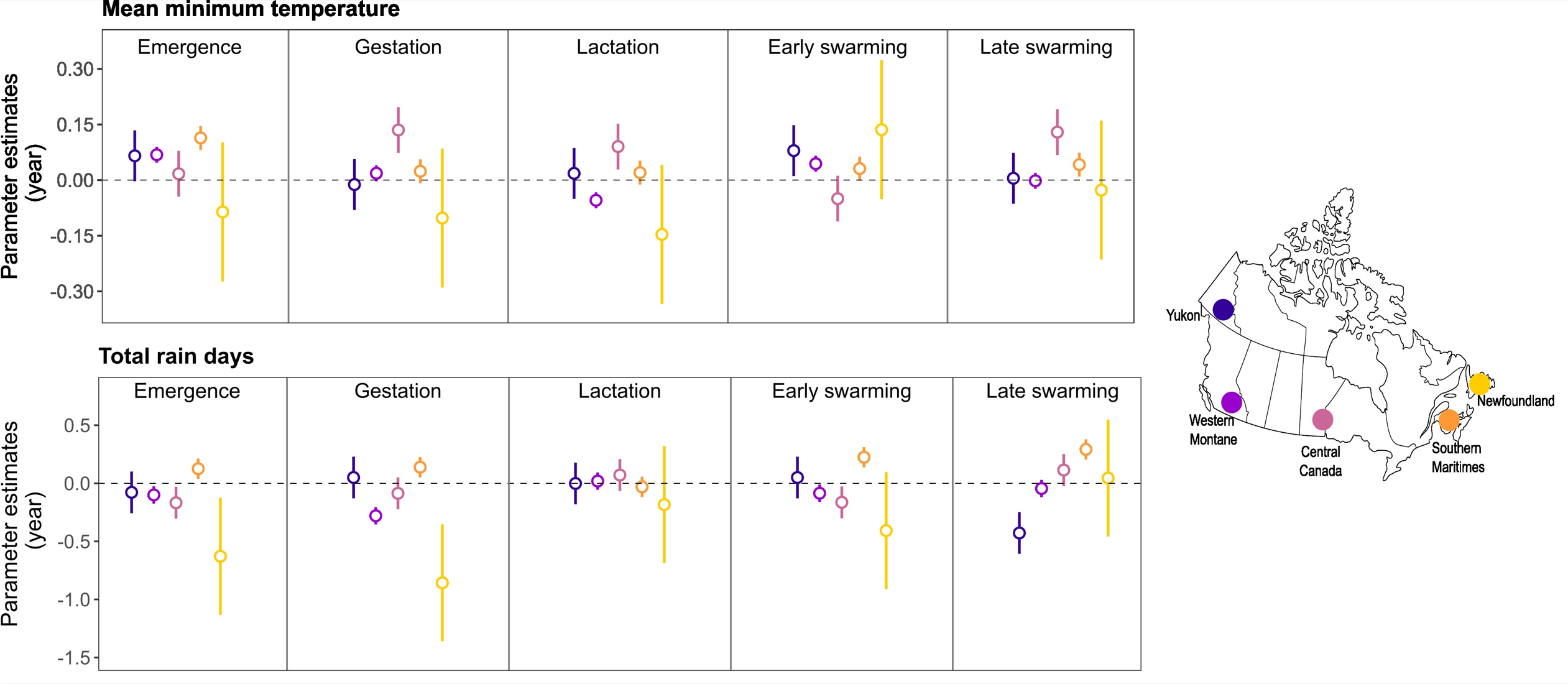


**Figure S3** Parameter estimates (open dots) and 95% confidence intervals (bars) from linear mixed-effect models predicting the effects of year on mean minimum temperature (top panel) and total rain days (bottom panel) for each active season phenophase of an adult female little brown bat (*Myotis lucifugus*) for each geographic region and respective study period. Confidence intervals are smaller than the point size when not visible. Mean minimum temperature and the total number of rain days increased over the study period when the parameter estimates are above zero, and decreased when estimates are below zero
